# DSF Core: Integrated Decision Support for Optimal Scheduling of Lifetime Extension Strategies for Industrial Equipment

**DOI:** 10.3390/s23031332

**Published:** 2023-01-25

**Authors:** Nikolaos Kolokas, Dimosthenis Ioannidis, Dimitrios Tzovaras

**Affiliations:** Centre for Research and Technology Hellas, Information Technologies Institute, 57001 Thessaloniki, Greece

**Keywords:** optimization, decision making, industrial equipment, manufacturing, circular economy, life extension strategy, cost modelling

## Abstract

This paper proposes a generic algorithm for industries with degrading and/or failing equipment with significant consequences. Based on the specifications and the real-time status of the production line, the algorithm provides decision support to machinery operators and manufacturers about the appropriate lifetime extension strategies to apply, the optimal time-frame for the implementation of each and the relevant machine components. The relevant recommendations of the algorithm are selected by comparing smartly chosen alternatives after simulation-based life cycle evaluation of Key Performance Indicators (KPIs), considering the short-term and long-term impact of decisions on these economic and environmental KPIs. This algorithm requires various inputs, some of which may be calculated by third-party algorithms, so it may be viewed as the ultimate algorithm of an overall Decision Support Framework (DSF). Thus, it is called “DSF Core”. The algorithm was applied successfully to three heterogeneous industrial pilots. The results indicate that compared to the lightest possible corrective strategy application policy, following the optimal preventive strategy application policy proposed by this algorithm can reduce the KPI penalties due to stops (i.e., failures and strategies) and production inefficiency by 30–40%.

## 1. Introduction

The European remanufacturing industry is estimated to have a total turnover of EUR 29.8 billion with 190,000 employees. Provided adequate support from public authorities, remanufacturing could reach up to EUR 90 billion and an associated employment of 600,000 by 2030 [1]. According to [2], the Europe Maintenance, Repair and Overhaul (MRO) distribution market size was estimated at USD 202.88 billion in 2021 and is expected to grow at a compound annual growth rate (CAGR) of 2.8% from 2022 to 2030. In addition, the manufacturing of machinery and equipment is one of the most competitive and largest manufacturing segments in Europe. Furthermore, based on [1], remanufacturing and refurbishment can contribute significantly to the well-being in Europe, as they are important lifetime extension strategies of resource-efficient manufacturing. By keeping components and their embodied material in use longer, significant environmental benefits can emerge. Less energy and fewer material resources are used, and less waste is created when products and components are used again instead of only recycling the materials. Opportunities for highly skilled jobs and economic growth would also arise; according to a recent study conducted by the European Remanufacturing Network Project, remanufacturing in Europe was assessed to reach about EUR 30 billion in 2015 and has been expected to triple up to about EUR 100 billion in Europe until 2030, employing over 500,000 people.

An electromechanical machine near its end of life (EoL) can no longer perform its required functions under the stated conditions, thus EoL decisions should be made well in advance in order to minimize the impact on production capacity [3]. One cost-effective decision is to extend the life of the machine via refurbishment or remanufacturing. However, so far there was no standard solution or even an established decision-making strategy for the refurbishing of manufacturing assets [4]. This raises the need for a Decision Support Framework (DSF) for a timely and accurate machine health forecast. There are three main challenges that need to be addressed during the decision-making process concerning the refurbishment of a machine. First, is the machine worth refurbishing? Second, what is the best time to perform refurbishment at the least cost? Finally, how should the machine be refurbished?

This paper deals with an algorithm responsible for providing recommendations to industrial end users (machinery operators and manufacturers) about the optimal lifetime extension strategy application policy for the analysed industrial equipment of the production line. This policy includes the selection of the strategies, the time of application of each and the relevant machine components. The application of this algorithm requires heterogeneous raw (sensorial or manually defined) or processed input data (past stops and downtime, degradation levels, failure probabilities, production efficiency, failure metrics, cost elements, etc.). Since many of these data may have been processed by third-party algorithms, this algorithm, which gathers all these inputs to provide integrated decision support, will be called “DSF Core” for simplicity. The DSF Core requires first training a DSF Core model based on (mainly manually defined) specifications of the production line to learn the optimal policy. Then, this model can be used (run) in order to simulate the status of the production line at every timestamp of a future time interval in terms of Key Performance Indicators (KPIs), numbers and time intervals of stops (failures and strategies) and production time percentage of each machine component under the optimal policy. It can also run real-time only to recommend strategies that should be used immediately. In both run scenarios, an initial (present) condition of the production line should be assumed. The actual condition may be inferred by information provided by the raw sensorial data and the data processed by third-party algorithms.

In particular, this algorithm may receive the following kinds of inputs. Unavailable processed inputs from third-party algorithms should be manually defined, be assigned some default values or be simulated using random generators.

- The direct sensorial input needed is the historical and real-time information about analysed stops (failures, strategies), i.e., start-end time, related machine component and reason for each instance. Usually, the aforementioned stop information cannot be recorded directly thanks to sensorial data, may be only manually registered, may have not been performed at all or performed incompletely or inaccurately. In these cases, the stop intervals should be identified based on the behaviour of other sensorial time series (e.g., based on zero or absent values), whereas failure diagnosis algorithms should infer the related component and stop reason, especially in the case of failures accompanied by some anomaly. Failure diagnosis may be performed, e.g., using failure mode and effects analysis [5] or probabilistic graphical models [6] [such as Bayesian methods [7] in general or the time-dependent dynamic Bayesian networks [8]].
- From predictive maintenance algorithms, such as those described in [9,10], the DSF Core needs the real-time failure probabilities for each failure type for a particular forecasting horizon, computed based on sensorial data.
- From degradation models, the DSF Core may receive the parameters of the Weibull distributions characterizing the lifetime of machine components with respect to various failure types. In relation to this, it may also receive real-time equivalent age and load information, which assess the respective degradation levels and degradation speeds, in accordance with the generic references [11,12]. There are also references about the reliability of specific common machine components considered in this work, such as bearing [13] and sample-detector [14].
- Thanks to extensive cost information, directly provided as input or computed based on [15] or by the DSF Core itself, the algorithm is able to perform short-term as well as long-term (using Monte Carlo simulation) assessment of the economic KPI, considering costs and profit both during production and during voluntary (strategies) and involuntary stops (failures).

The DSF Core, as well as third-party algorithms which may provide potential useful input to it from each of the above kinds, have been developed within the context of the RECLAIM project (see Funding section). The algorithm was successfully applied to three pilot industries involved in this project: GORENJE (white goods manufacturer, the white enamelling line of which is studied in this paper), HWH (producer of control systems in the welding sector) and ZORLUTEKS (cotton textile manufacturer). A couple of indicative useful DSF Core models from each pilot will be discussed in this paper.

Particularly, the remainder of the paper is organized as follows. The rest of Section 1 presents the related work found in the bibliography and the contribution of this work, Section 2 describes the generic methodology of the algorithm, and Section 3 deals with the implementation of the algorithm per pilot for various machinery components. More especially, Section 3 includes the input and results related to the trained DSF Core models, as well as execution examples of these models with simulation of the production line life cycle under the optimal strategy application policy and the relevant impact on KPIs. The results are also discussed in the same section. The deliverable is concluded in Section 4.

The lifetime extension (circular economy) and conventional (linear economy) strategies applied during the machinery life cycle which are considered by the DSF Core were defined in [16], andwere also instantiated per pilot during workshops. References  [3,17,18,19,20] include figures that mention such machinery as product and show the flow among states of the machinery during its life cycle based on the applied strategies.

### 1.1. Related Work on Decision Support and Optimization Plans

Table 1 summarizes the bibliographic findings about the candidate decisions proposed by decision support frameworks in manufacturing, the KPIs evaluating decision support optimization criteria, the methods used and the required input data to the optimization algorithm. A particularly systematic classification of KPIs appears in [21,22], based on which the KPIs of this work were coarsely classified as economic and environmental, as explained later in detail. In the former work, the KPIs are first scaled in the range 0–100 and then weighted based on factors defined by stakeholders, and it is asserted that they should also be determined with the help of historical data when possible. Unlike other available methodologies, the latter paper considers all the interests of the stakeholders involved in the product life cycle, who also define the KPI weighting factors based on their objectives, which generally vary and may even be conflicting.

### 1.2. Related Work on Cost Analysis and Cost Modelling Tools

Since the economic KPI is the most important one, a cost modelling and financial analysis methodology should definitely be embedded into a decision support framework for sustainable refurbishment and remanufacturing of used industrial equipment. The DSF Core relies on [15] to estimate the costs during stops and calculates on its own the cost and profit during production. Based on the above, using a Monte Carlo simulation including discrete event simulation, it is able to further estimate economic KPI. Table 2 summarizes the information in indicative bibliographic references about the types of estimated costs, the methods used and the required input data. Most of these references are related to additive manufacturing. A more extensive bibliographic study on cost modelling and analysis is included in [15].

The above studies consider cost only during specific stages of products life cycle. However, insightful decision making requires cost evaluation during the whole life cycle. The review in [68] examines life cycle cost (LCC) studies, along with the complementary life cycle assessment (LCA), which corresponds to the environmental factors, and the integration between the two. According to that review, there is no general standard for the application of LCC; there is just ISO 15686-5 for buildings and built assets. Although summing up costs per functional unit is a common practice, the detailed cost elements depend on the objectives of each study. LCA, along with its integration with LCC, is discussed in the next heading.

### 1.3. Related Work on Life Cycle Assessment and Integration with Life Cycle Cost

As mentioned above, LCA is the evaluation of environmental KPIs throughout a product’s life cycle. Thus, this subsection complements the previous one, which examined the economic KPI.

LCA is performed at three system boundary levels with corresponding considered impacts as mentioned below [69]:

- *Cradle-to-gate:* raw material extraction and material production until the exit of the product from the factory;
- *Cradle-to-grave:* raw material extraction, material production, exit of the final product from the factory, as well as the use, demolition and waste phases;
- *Cradle-to-cradle:* raw material extraction, material production, exit of the final product from the factory, as well as the phases of use, demolition, waste, recycling and extensive reuse of the waste.

Our work evaluates the economic and environmental KPIs from the viewpoint of the user of the industrial machinery throughout a representative period of production with intermediate failures and lifetime extension strategies regarding this machinery.

The particular challenge posed by the environmental factors is the diversity of their measurement units. According to [70], environmental factors generally concern resource consumption and greenhouse gas emissions. A finer classification of the environmental factors follows below:

- air (global warming potential, ozone layer depletion potential,…);
- Water (water depletion, eutrophication potential,…);
- Energy (cumulative energy demand, fossil fuel depletion,…);
- Soil (land occupation, acidification potential,…);
- Human (human toxicity potential from chemicals and pollutants released,…);
- Other (minerals depletion, solid waste,…).

As admitted in this reference, although there are standards for the application of LCA, they are too general, giving much freedom regarding the definition of scopes, boundaries and functional units.

Examples of methods for integrating the environmental factors within a common environmental indicator are mentioned in [69,70] are CML 2001, CML 2002, EDIP97–EDIP2003, Tool for Reduction and Assessment of Chemicals and Other Environmental Impacts, ReCiPe, Eco-indicator 99, Environmental Priority Strategies 2000, Impact 2002+ (applied, e.g., in the industry-related paper [71]), LIME, LUCAS, Swiss Ecoscarcity and the Methodology Study for Ecodesign of Energy-using Products. However, the comparison of the environmental factors with the economic KPI is another issue. For this reason, a good practice is the monetization of environmental impacts [68], which was adopted in our paper. Another alternative is the use of fuzzy logic and the Likert scale [68,72] to qualify the heterogeneous factors in a common scale for comparison. However, such an approach has the drawback that the qualitative evaluation is subjective, and the evaluation accuracy is partly lost due to discretization. What is more, in our paper, the upper limits of the original individual KPIs are highly dependent on the lifetime extension strategy application policy, which would make the mapping to the Likert scale even more questionable and subjective. Finally, there are some hybrid approaches combining LCA with exergetic analysis with the use of the second law of thermodynamics, but they also suffer from the lack of appropriate indicators and a well-established set of calculations, as well as the lack of complete and updated data to overcome the uncertainty [73].

According to [69], many input data are needed to apply LCA. In the RECLAIM project, most sensorial data became available recently, and a systematic LCA has not been performed within the context of the DSF Core, but it will be conducted by another tool afterwards. Instead, the DSF Core at this stage relies on static specifications, assumptions and simulations to evaluate the environmental impacts within a sufficiently large future interval.

As is easily observable from the literature review conducted on LCA, the detailed environmental elements which are worth considering are use-case-specific. The aforementioned work in [70] examines textile production, so it is comparable and quite in line with the evaluation of environmental KPIs in our paper for the ZORLUTEKS pilot. However, related work on LCA for white goods manufacturing (GORENJE pilot) and friction welding (HWH pilot) was not found. There is just related work on welding [74]. In our work, each pilot selected for itself the most important environmental elements and the desired improvements in them to be achieved during the RECLAIM project. These were classified for the DSF Core based on [21] and according to their measurement units. LCA has also been applied to other industrial use cases, such as mass timber [75] and silicon photovoltaic modules [76] production, as well as in other settings.

### 1.4. Contributions of This Work beyond the State of the Art

The mathematical formulation of the optimization problem to be solved by the DSF Core algorithm in RECLAIM was inspired mainly by the MCDA approach of [21], which was also taken into account by the authors in their previous work on demand-side management [77] and is suitable for integrating economic and environmental KPIs [68]. However, this method in those papers is not combined with life cycle evaluation of KPIs, which enables decision support considering the strategy application policy impact on their values both during the application of strategies and in the long run. In general, as mentioned in the review of [25], big data analysis for smart decision making for the whole life cycle of a production line considering multiple objectives is rarely performed, so the current work may support progress in this research direction. In contrast to the other relevant methods found in the literature [23], which balance heterogeneous KPIs subjectively, this work introduces a method for automatic objective balancing of KPIs measured in different units by converting all environmental units to economic units. In addition, this paper demonstrates that, thanks to life cycle evaluation of KPIs and Monte Carlo simulation, decision support may be provided not only for the present, but for any timestamp of the simulation interval by estimating when each strategy will be favorable in the future under a specific optimal application policy. Since the evaluation of the objective function in this work is time-consuming and the value of the function depends on its arguments only within a subset of its domain, a typical optimization method, such as MINLP [58,59], is inappropriate and intractable. Thus, a technique for smarter selection and updating of the decision variables is introduced, so that the function is optimized with the fewest possible evaluations. The DSF Core supports health-based recovery planning at the component, machine and production line level while considering economic and environmental effects. Instead, other studies, such as [57], disregard the environmental factors and only consider the economic KPI or vice versa [56]. The DSF Core aims at helping the machinery operators and manufacturers make efficient EoL decisions at different service and life periods. More especially, it considers failure probabilities estimated based on both real-time sensorial data and the degradation level of components, as well as detailed cost modelling and financial analysis computations. In all cases, it considers dependencies among different machine components in terms of failures and operational efficiency in contrast to related references considering a single component, such as [27]. Furthermore, the quantitative optimization method used in this paper seems to be more suitable for the problem solved than qualitative approaches [18,28,30,61], since the latter involve some information loss due to uncertainty.

The cost modelling and analysis method of this paper also goes beyond the state of the art because apart from the static specifications it also supports the consideration of the (near-)real-time condition of the production line, expressed by stops and the degradation level of components. Furthermore, as mentioned, the Monte Carlo simulation method developed in this work enables a relatively accurate cost estimation for every timestamp of a future time interval as well, and by extension a life cycle estimation of the economic KPI. This also applies to the other KPIs when considered. The other KPIs are generally simpler and more straightforward to compute, both during stops and during production, as shown later. Previous similar studies generally do not consider life cycle cost dependent on the health status of the production line, only some constant costs during production [63,64,66] or during lifetime extension strategies, such as manufacturing [62,65].

Finally, it needs to be clarified that some of the above papers are not fully comparable with the current one because they solve a somehow different problem. More especially, [23,24,58,59,67] make decisions based on supply and demand rather than the health of the equipment, whereas other references [17,60] consider a single phase of the life cycle, namely design and disassembly.

## 2. Materials and Methods

The optimization of the DSF Core algorithm surrounding strategies recommended for machines and machine components of a production line, as explained in Section 1, is based on a mathematical programming problem of minimizing a non-linear scalar objective function. This function quantifies a mean (average) total KPI over a sufficiently large simulation interval. At every timestamp of this interval, obtained using a constant sampling rate, the total KPI is the weighted sum of independent individual KPIs with different measurement units. In the following, the mean individual KPIs and the mean total KPI will be scaled so that they correspond to average yearly values. Coarsely, based on [21,70] and the RECLAIM pilot goals, the individual KPIs in this project may be categorized as economic (including several cost elements all measured in Euros) and environmental (including the sub-categories about resource use and emissions). As described later in detail, every individual KPI at a particular timestamp is computed based on the condition of the production line at that moment, considering whether production is active or not, potential stops (failures or strategies) occurring and the production inefficiency due to degradation of machine components. Failures are simulated using discrete event simulation, considering short-term failure probabilities based on degradation of the respective components or other reasons. The failure probabilities within the simulation interval are randomly generated from particular probability distributions, and, usually, consecutive failure probability values for the same failure type are correlated. The evolution of the production line condition (and thus the KPIs) is estimated iteratively.

Every machine is assumed to consist of at least one component, particularly, at most one static component and potential movable components in the sense that a movable component can be changed without replacing the rest of the machine, whereas the change of the static component signifies the change of the machine. For the static component, refurbishment is the only candidate circular economy strategy that will be discussed in this paper. The definition of refurbishment is mentioned in [16] as follows: “Refurbishment means restoring an old product and bringing it up to date. In general, refurbished products are upgraded and brought back to specified quality standards or satisfactory working and/or cosmetic conditions and have to fulfill extensive testing. Occasionally, refurbishing is combined with technology upgrading by replacing outdated modules and parts with technologically superior ones”. For a movable component, replacement should always be a candidate strategy, whereas maintenance is also a possible lighter strategy, with one or more alternatives. In the following, wherever it is mentioned that the machine fails or is refurbished, it will be implied that this happens to the static component. Refurbishment of the static component can be combined with strategies to the movable components in multiple ways. More concretely, a strategy in a component generally does not imply the (in)ability to simultaneously apply a strategy in other component(s), with the following exceptions, stemming from the definition of refurbishment:

- During refurbishment of a static component, maintenance of movable components of the same machine is impossible. In this case, the movable components may either be replaced or remain as they are. For some movable components, replacement may be compulsory in this case.
- The reverse is true. During maintenance of a movable component, refurbishment of the same machine is impossible.

The optimization consists of two main phases:

1 Pre-optimization for corrective strategies σ: Assuming initially that a strategy is always applied to a component and only as soon as possible after the component has failed, this phase finds the optimal strategy for each component after its failure. The initial solution considers the lightest compulsory corrective strategy for each failure type. The lightest compulsory corrective strategy to be applied after a failure is defined by the user, as explained later. For every failure type, the possible corrective strategies are the possible preventive ones which are not lighter than the above.
2 Main optimization for decision variables θ related to:
  - *Short-interval average modified KPI threshold per component and strategy applicable to it:* The strategy is recommended and applied when the average total modified KPI (based on a modification described later) exceeds the threshold. If the threshold is exceeded for multiple strategies for the same component simultaneously, the heaviest one is selected. (If this happens for multiple maintenance alternatives simultaneously, the one defined first by the user is selected.) If strategies and failure fixations may take place only during working hours or the present time is non-working time, the strategy is assigned the status “urgent” and is applied as soon as possible. Otherwise, it is assigned the status “non-urgent” and is also applied as soon as possible, but only during non-working hours in order not to interrupt the production, which would cause indirect economic loss, since fewer parts would be produced due to the additional downtime. Exceptionally, if refurbishment for the static component has been chosen, any proposed strategy for movable components is assigned the same urgency status as refurbishment.
  - *Failure probability threshold per component and failure type:* The lightest strategy applicable to the component is recommended and applied when the probability of any failure in this component exceeds the threshold. If strategies and failure fixations may take place only during working hours or the present time is non-working time, the strategy is considered “urgent”. Otherwise, it is considered “urgent” (instead of “non-urgent”) if and only if the failure probability until the next non-working timestamp is much higher than the failure probability at the next timestamp, also considering the impact of the strategy on the downtime of components and the time distance until the next non-working timestamp, based on the formula
    (1) $$P_{long}\geq 10^{\Delta p}nP_{short},$$
    according to the following notation:
    – $P_{short}$: probability for failure of the component in question to happen at the next timestamp, according to the considered (constant) sampling step;
    – $P_{long}$: probability for failure of the component in question to happen at the next non-working timestamp;
    – *n*: number of time steps until the next non-working timestamp;
    – Δp: difference in percentage of non-working machine components if the strategy is applied and not.
    For a particular component and failure type combination, due to high time complexity, the relevant computations take place only at the first consecutive working timestamp for which the computation of urgency makes sense. For the other timestamps among the above, the same urgency is defined. In addition, when the status “non-urgent” is assigned to the recommended strategy at the last working timestamp of a working interval, the strategy is recommended with status “urgent” at the next timestamp (i.e., the first of the next non-working interval). This happens even if the respective failure probability threshold with respect to the load during non-working hours at that timestamp is also exceeded.
    Exceptionally, if refurbishment of the static component has been chosen, any simultaneously proposed strategy for movable components is assigned the same urgency status as refurbishment.
  - *Corrective strategies “actuators” per component and failure type:* Binary variables determining if the optimal corrective strategy for the component as found during the pre-optimization phase will indeed be applied as soon as possible (with status “urgent”) after the component fails.

The objective function quantifying the mean yearly total KPI is defined as

(2) $$g\left(\sigma ,\theta \right)=\frac{\mathrm{number}\;\mathrm{of}\;\mathrm{yearly}\;\mathrm{timestamps}}{L}\sum_{i=1}^{L}\sum_{c=1}^{C}\sum_{k\in K}w_{k}K_{c}^{k}\left(i,\sigma ,\theta \right),$$

with the following notation:

- $K_{c}^{k}\left(i,\sigma ,\theta \right)$: instant individual KPI of type *k* of component *c* at the *i*-th timestamp of the simulation interval under corrective strategies σ and decision variables θ;
- $w_{k}$: weighting coefficient of respective KPI $K_{c}^{k}\left(i,\sigma ,\theta \right)$.

Complex assumptions about the behavior of the KPIs under various circumstances (e.g., regarding their dependence on the degradation level of machine components) need to be taken into account, as will be explained further below. Thus, the objective function of this optimization is difficult to define as an analytic function, which would enable its optimization with the help of its derivatives with respect to its continuous input variables. Moreover, the computation of an exact solution using dynamic programming has too high a time complexity. Furthermore, since some stop periods have relatively short duration and some stops rarely occur, to reliably estimate individual KPIs, a long simulation time interval with many timestamps is needed, which would require many operations for iterative calculations. In this work, we perform a Monte Carlo simulation including discrete event simulation to evaluate and optimize *g* first with respect to σ (pre-optimization) and then with respect to θ (main optimization), thus finding the optimal corrective and preventive policy for applying lifetime extension strategies. To decrease computational complexity as much as possible, a specific greedy optimization algorithm was introduced, thanks to which the number of objective function evaluations is much lowith respect toan if a common non-linear optimization method was applied. The optimization of *g* with respect to σ and θ, shown in Algorithm 1 and Algorithm 2, respectively, will be called “training”.

The inputs of Algorithm 1 follow below. Those with an asterisk are directly defined by the user, and thus they are discussed in detail in Section 2.1. The rest are computed by or included in the DSF Core itself.

- Number of strategy types;
*Failure types;
*Lightest compulsory corrective strategies;
- Objective function *g* evaluated based on simulation;
*Stop types corresponding to decision variables to be excluded from the optimization (these are not considered in Algorithm 1);
- Function sorting strategy types based on their effect (for comparisons with the lightest compulsory strategies to determine which strategies are applicable after each failure).

The outputs of Algorithm 1 are the optimal σ and corresponding *g* value.
**Algorithm 1** Algorithm for pre-optimization for corrective strategies σ1:compute *g* without applying strategies in preventive way, for σ corresponding to applying the lightest compulsory corrective strategy corresponding to every failure2:epoch←03:**while** no termination criterion applies (epoch number/no improvement within 1 epoch) **do**4:    **for** every combination of component and failure type **do**5:        **for** every corrective strategy applicable to the component after such failure **do**6:           compute *g* if not computed with same arguments yet7:           **if** the value of *g* is better than the optimal found so far **then**8:               update optimal σ and optimal *g* value9:           **end if**10:        **end for**11:    **end for**12:    epoch←epoch+113:**end while**

Strategies are never recommended in a preventive way when the KPI and failure probability thresholds are high enough so they are never exceeded. Therefore, high values are initially chosen. For probabilities, values equal to 1.1 are set. For KPIs, a single maximum KPI value of $10^{4}$ is considered in this paper. In addition, initially, all corrective strategies “actuators” are active, i.e.,strategies are always applied after relevant failure instances. This is important because if the optimization starts from a really bad solution some component will never undergo any strategy.

The maximum number of optimization epochs will always be set to 10 but will never be reached, as discussed later.

The main inputs of Algorithm 2 follow below. Those with an asterisk are directly defined by the user; thus, they are discussed in detail in Section 2.1. The rest are computed by or included in the DSF Core itself.

*Strategy types;
*Failure types;
*Lightest compulsory corrective strategies;
- Objective function *g* evaluated based on simulation;
*Stop types corresponding to decision variables to be excluded from the optimization (these are not considered in Algorithm 2);
- Function finding every time the next θ for which *g* is to be evaluated;

The main outputs of Algorithm 2 are the optimal θ and corresponding *g* value.

The use of percentiles also plays an important role in the reduction of function evaluations because the KPI and failure probability thresholds are updated in such a way that the numbers of instances from some stop types are significantly affected, also considering that the objective function is independent of the decision variables in some regions. The percentiles are updated every time a new solution is found because the distribution of KPIs and failure probabilities have been affected by the updated policy.
**Algorithm 2** Algorithm for main optimization for decision variables θ1:initialize σ as the optimal one found during pre-optimization2:**for** every KPI time series (each corresponding to a component—strategy type combination) and failure probability time series (each corresponding to a component—failure type combination) **do**3:    compute the 100(1−nr)% percentiles of its instant values observed during the computation of *g* for the optimal pre-optimization solution, with resolution *r* [n∈N,1−nr∈[0,1]]4:**end for**5:**while** no termination criterion (maximum epochs reached or *g* not improved within 1 epoch) applies **do**6:    **for** every decision variable **do**7:        **if** the decision variable corresponds to KPI threshold or failure probability threshold **then**8:           compute *g* for the closest lower and higher values for this threshold corresponding to respective percentiles if not computed with same arguments yet9:        **else**(decision variable corresponds to corrective strategy “actuator”)10:           compute *g* for the other value of this binary “actuator” if not computed with same arguments yet11:           **if** the value of *g* is better than the optimal found so far **then**12:               update optimal θ and optimal *g* value13:               update KPI and probability percentiles based on new optimal solution, keeping also the initial thresholds as candidate values14:           **end if**15:        **end if**16:    **end for**17:    epoch←epoch+118:**end while**

### 2.1. Input Parameters to the DSF Core Training

*Machines of the production line studied by the DSF Core model, components of each machine, as well as potential stop (strategy and failure) types corresponding to each component:* Only the names of machines and components considered by the DSF Core model in question should be defined. If other machines and components are considered by other DSF Core models (which should be considered in the case of many independent or similar machines/components, to reduce computation time, since time complexity is quadratic with respect to the number of stop types, and to avoid multiple trainings for similar components), just their number needs to be mentioned. In this paper, it is assumed that when a component stops, this causes the stop of production of the whole production line. However, the other components do not fail, i.e., they are not damaged. When the originally stopped component is restored, the others are also assumed as operational. Furthermore, no strategy is applicable to a failed component until it is fixed, even if the strategy is proposed by the DSF Core. Production is quantified per component and may stop due to the aforementioned reasons. Finally, it needs to be noted that no multiple failures may occur simultaneously in the same component. Even when mentioned that a failure of one type immediately causes a failure of another type (as discussed later), the latter starts at the next timestamp after the end of the former.

*Components to be always replaced along with refurbishment:* These should be defined for every machine for which refurbishment has been selected as a candidate strategy. Selected components must always be replaced along with refurbishment, but they may also be replaced at other times.

*Stop types corresponding to decision variables to be excluded from the optimization;* It makes sense to exclude failures which never happen (i.e., never cause downtime) but lead to degradation-based inefficiency during production, as expressed with some KPI(s). It also makes sense to exclude strategies that should be applied due to failure or high failure probability, but not due to KPI-expressed inefficiency.

*Lightest compulsory corrective strategies:* For every failure type, the lightest compulsory corrective strategy to make the component work again should be defined. It is possible not to select any strategy for some failure type. In this case, it is assumed that there is also the option just to fix the failure without improving the respective equivalent age (which quantifies the degradation level of the component with respect to this failure type, as discussed below). Maintenance alternatives belonging to the same high-level strategy type are assumed as sorted previously by the user in ascending order of effect.

*Failure type dependencies:* Based on prior pilot knowledge, the dependent failure types are considered by the DSF Core. In this paper, a failure type $f_{2}$ is dependent on another failure type $f_{1}$ if a failure of type $f_{2}$ occurs immediately after a failure of type $f_{1}$.

*Sampling step:* The selected value is usually close to the minimum stop duration, as defined later, to ensure both sufficient time granularity and tractable time and memory complexities.

*Working hours (from which the gross profit rate is computed):* These are assumed as dependent on the day of week.

*Indication if strategies and failure fixations may take place only during working hours:* Yes/no.

*Simulation interval length:* The selected value is close to 30 times the maximum Mean Equivalent Time To Failure (METTF), discussed later, to ensure both that enough instances from each stop type occur within the simulation interval and that the time and memory complexities are not too high.

*Remaining equivalent age after strategy compared to before (percentage):* Each simulation for training starts assuming that the production line is new. As the time passes, the components are gradually aging with respect to their various failure types. Depending on the component and the failure type, aging speed generally (which will be called “load” to follow the degradation modelling terminology) depends on whether the component is working, and it is also generally affected by the way in which it is working, as described by available process variables. The ages of components with respect to their failure types consider these factors, so they are called “equivalent ages”. The equivalent age of a component with respect to a particular failure type partly affects the occurrence probability of a failure of this type, based on Weibull distributions, as explained later. The percentage of equivalent age that a component has with respect to each of its failure types after a strategy compared to before is assumed as dependent on the component, the strategy type and the failure type.

*Load specifications:* The load specifications follow below:

- *Range:* The range is per component and failure type during periods with and without production. The load starts from a random number within the range and evolves according to the maximum absolute first-order difference within 1 day defined below.
- *Maximum absolute first-order difference within 1 day:* This is a single value divided by the average number of timestamps per day to compute the maximum absolute change between two consecutive timestamps, based on the considered sampling step.
- *Multiplier during production due to high failure probability until the next timestamp:* This is a per ordered pair of possibly equal failure types, where the first corresponds to the multiplier and the second to the failure probability. The probability is considered as high (resulting in the multiplication of load by the multiplier) when it exceeds the corresponding degradation-based probability of failure until the next timestamp at 90% of the respective METTF (discussed later).

*KPI types considered:* The economic KPI is mandatory because it is the only one related to benefit, and it is measured in some currency unit.

*Measurement units of KPIs:* A measurement unit should be defined per KPI type.

*KPI weighting coefficients:* The environmental KPIs are monetized based on unit prices of environmental elements, but the user may define any other possible weights.

*Ideal average yearly values of KPIs:* “Ideal” means that the equipment always works as efficiently as when it was new, and all failures always have probability of 0, so there is no need for strategies. The yearly values within a certain year are computed by summing up the instant values within this year. All KPIs are assumed to have a neutral value of 0, while the respective components are not producing. The ideal average yearly values of KPIs are assumed to depend only on the KPI type *k*. The values to be defined should correspond to the whole production line, including potential machine components absent from the current DSF Core model but considered by other DSF Core models.

*KPIs evolution and dependencies:*

During production, the component individual KPI $K_{c}^{k}\left(i,\sigma ,\theta \right)$, present in the objective function, is defined as the mean of failure-type-specific KPI sub-values $K_{c,f}^{k}\left(i,\sigma ,\theta \right)$ for all failure types *f* of the component *c*. During production, each such sub-value is assumed to equal an equivalent-age-based exponential inefficiency penalty for the respective KPI type *k*, component *c* and failure type *f*, i.e.,

(3) $$K_{c,f}^{k}\left(i,\sigma ,\theta \right)=v_{k}+b_{k,c}^{s_{c,f}\left(i,\sigma ,\theta \right)}-1.$$

In the above formula, $s_{c,f}\left(i,\sigma ,\theta \right)$ stands for the equivalent age of component *c* with respect to failure type *f* at time *i* given policies σ and θ. In addition, $v_{k}$ is the ideal value of KPI *k* divided by the average yearly working timestamps (i.e., within the potential working hours of the production line, whether or not there are any irregular stops among the aforementioned) and the number of machine components (with $\sum_{k\in K}w_{k}v_{k}<0$, since the production of the production line is profitable only for negative *g*) and $b_{k,c}>1$. Because it is not straightforward to define $b_{k,c}$ directly, the user defines the inefficiency $\left(b_{k,c}^{s_{c}^{*}}-1\right)\cdot \frac{1\mathrm{day}}{l}$, where $s_{c}^{*}$ (equivalent age of component *c* corresponding to the inefficiency) is either manually defined by the user or automatically selected as 90% of METTF of component *c*. The METTF of a component is defined as the harmonic mean of METTF values of its failure types, with respect to either the time-to-failure distributions based on equivalent age or the failure probability distributions due to process-data-related reasons (see later).

Then, the auxiliary KPIs ${\hat{K}}_{c,f}^{k}\left(i,\sigma ,\theta \right)$, aiming at distributing the KPI sub-values $K_{c,f}^{k}\left(i,\sigma ,\theta \right)$ among the failure types of the respective component, are computed as

(4) $${\hat{K}}_{c,f}^{k}\left(i,\sigma ,\theta \right)=\frac{K_{c,f}^{k}\left(i,\sigma ,\theta \right)}{|F_{c}|},$$

where $F_{c}$ is the set of possible failure types of component *c*.

If a failure *f* starts at time *i* in component *c*, its direct impact on each KPI as defined by the user (see later) is added to both $K_{c,f}^{k}\left(i,\sigma ,\theta \right)$ and ${\hat{K}}_{c,f}^{k}\left(i,\sigma ,\theta \right)$. If a strategy *s* starts at time *i* in component *c*, its user-defined direct impact on each KPI as defined by the user is added initially to $K_{c,f}^{k}\left(i,\sigma ,\theta \right)$. [The fact that it is not added to ${\hat{K}}_{c,f}^{k}\left(i,\sigma ,\theta \right)$ is compensated later, as will be explained right after.] Apart from the above, at the other timestamps without production from component *c*, it is

(5) $$K_{c}^{k}\left(i,\sigma ,\theta \right)=K_{c,f}^{k}\left(i,\sigma ,\theta \right)={\hat{K}}_{c,f}^{k}\left(i,\sigma ,\theta \right)=0\forall k\in K,f\in F_{c}.$$

Let ${\tilde{K}}_{c,s}\left(i,\sigma ,\theta \right)$ be the instant total modified KPIs for every component *c* and strategy *s*, based on which the average values compared to the respective thresholds are computed according to the short interval lengths of 1D, 7D and 30D for maintenance, replacement and refurbishment, respectively. If strategy *s* does not start at time *i* in component *c*, these are defined as

(6) $${\tilde{K}}_{c,s}\left(i,\sigma ,\theta \right)=\sum_{f\in F_{c}}\left(1-q_{c,h(s),f}\right)\sum_{k\in K}w_{k}{\hat{K}}_{c,f}^{k}\left(i,\sigma ,\theta \right),$$

where $q_{c,h(s),f}$ is the remaining equivalent age percentage with respect to failure of type *f* after the application of a strategy belonging to the high-level group h(s), including *s* to *c*. The rationale for considering the factors $1-q_{c,h(s),f}$ is that the more a strategy reduces in percentage the equivalent age of a component with respect to some of its failure types, the higher the importance of reducing inefficiency, as quantified by the ${\hat{K}}_{c,f}^{k}\left(i,\sigma ,\theta \right)$ values for every *k*. This way, the inefficiency is mapped from the failures to the strategies, and there is no need to consider combinations of strategies and failure types in the thresholds, which would significantly increase the number of decision variables.

If strategy *s* starts at time *i* in component *c*, the above value is increased by the weighted sum of direct impacts of the strategy on the KPIs, which had previously been added only to $K_{c,f}^{k}\left(i,\sigma ,\theta \right)$ and not to any ${\hat{K}}_{c,f}^{k}\left(i,\sigma ,\theta \right)$, for every *k*.

If strategy *s* ends in component *c* at time *i*, the values of ${\tilde{K}}_{c,s^{*}}\left(i,\sigma ,\theta \right)$ for every equivalent or lighter strategy $s^{*}$ and every j≤i are not considered afterwards in the average values compared to the respective thresholds, based on the assumption that strategy *s* addressed successfully, and better than $s^{*}$ could do, any problem observed before through the above KPIs.

As mentioned above, some machine components may not be considered by the DSF Core model in question but may be considered by other DSF Core models. Then, the downtime impact of stops of considered components on out-of-model ones is taken into account by uniformly charging the potential KPI differences in the out-of-model components (comparing with the ideal values) due to stops of considered ones to the respective KPIs of all considered components at the same timestamps.

*Stop duration Weibull distribution parameters:* The duration of each stop type is assumed to follow the 2-parameter Weibull distribution with user-defined scale η and shape β parameters dependent on the stop type. These can be selected based on the coefficient of variation (CV) and mean (MSD) of stop duration, since theoretically they depend on each other based on the following well-known 1-1 functions:

(7) $$CV=\frac{\sqrt{\Gamma \left(1+2/\beta \right)-\Gamma^{2}\left(1+1/\beta \right)}}{\Gamma (1+1/\beta )},$$

(8) MSD=ηΓ(1+1/β),

where Γ denotes the gamma function. The parameter values may rely on prior domain expertise or analysis of historical data. Given that duration S=s has already passed, the probability distribution of the remaining duration *U* is computed as follows, considering that V:=S+U follows the Weibull distribution with scale η and shape β:

(9) $$P\left(U>u|S=s\right)=P\left(V>s+u|V>s\right)=\frac{P(V>s+u)}{P(V>s)}=\frac{e^{-{\left(\frac{s+u}{\eta }\right)}^{\beta }}}{e^{-{\left(\frac{s}{\eta }\right)}^{\beta }}}=e^{{\left(\frac{s}{\eta }\right)}^{\beta }-{\left(\frac{s+u}{\eta }\right)}^{\beta }},$$

where in every case *P* denotes the probability of the event in the brackets, and u≥0. For the purpose of simulation, the probability P(U<l|S=s) (i.e., for the next time step, which has length *l*) is computed. Usually, the sampling step is selected as comparable to (rather than negligible with respect to) the minimum MSD to balance time granularity and computational (or also memory) complexity. Thus, to avoid bias in the estimated downtime, when U<l given that S=s based on the above probability and the random number generator, it is assumed that

(10) $$U=\frac{l}{2},$$

and the relevant effects on KPIs apply accordingly, based on the above.

*Equivalent Time To Failure (ETTF) distribution parameters based on equivalent age:* As mentioned above, the equivalent age of a component partly determines the probability of future failures in this component. Like stop duration, ETTF follows the 2-parameter Weibull distribution for the purpose of simulation, but it applies only to failures and also considers load depending on the usage of the equipment during working hours apart from the working hours themselves for the measurement of time passed (hence the term “equivalent time”). Mathematically, equivalent time $t_{eq}$ passed within a regular time interval [0,t] is defined, as mentioned in [11,12], as

(11) $$t_{eq}=\int_{0}^{t}a\left(\tau \right)\;d\tau ,$$

where *a* denotes load, expressed as a function of the timestamp τ. Within short intervals, load can be assumed as nearly constant, so equivalent time may be approximated as

(12) $$t_{eq}=at.$$

This approximation is taken into account in the computation of the failure probability of each component within the next time step for each of its failure types given the component’s equivalent age similar to the remaining stop duration case. In cases of dependent failure types, according to the failure type dependencies discussed above, after a strategy happens, the Weibull parameters along with the equivalent age remain the same as those that would apply without considering the stop correlation.

*Mean Time To Failure (MTTF) values for process-data-related component failures and frequency of their probabilities’ change during production:* Apart from equivalent aging, component failures may also occur for other reasons, which may be captured by anomaly detection and predictive maintenance algorithms. For the purpose of simulation during training, depending on the failure type, the failure probability within the next time step is considered as a random variable from the Beta probability distribution β(α,β) (so that it ranges from 0 to 1), with probability density function $\frac{\Gamma (\alpha +\beta )}{\Gamma (\alpha )\Gamma (\beta )}x^{\alpha -1}{\left(1-x\right)}^{\beta -1},x\in \left(0,1\right)$. For a particular failure type of a component, this probability is not updated at every timestamp; it is always updated right after the end of a strategy applied to the same component, otherwise it is updated with a probability based on a user-defined frequency. The failure probabilities are considered only for working components, since it is assumed that a component may fail due to a process-data-related reason only while working (even if aged equivalently with respect to some failure types during periods without production by this component). The Beta distribution parameters are related with the MTTF, as well as the mean (MP) and coefficient of variation of the random variable (in this case, failure probability) ($CV_{P}$) based on the well-known formulas

(13) $$MP=\frac{1}{\mathrm{MTTF}}=\frac{\alpha }{\alpha +\beta },$$

(14) $$CV_{P}=\sqrt{\frac{\beta }{\alpha (\alpha +\beta +1)}},$$

where MTTF is measured in number of time steps based on the considered sampling step. The Beta distribution parameters may be defined as a function of MP and $CV_{P}$ by solving the above system:

(15) $$\alpha =\frac{1-MP(1+CV_{P}^{2})}{CV_{P}^{2}}=\frac{1-MP}{CV_{P}^{2}}-MP,$$

(16) $$\beta =\alpha \left(\frac{1}{MP}-1\right).$$

In the following, it will always be realistically assumed that α=0.1.

*Cost input:* This stop-related input must be either defined manually or computed by a third-party algorithm. The detailed methodology of estimating the various cost elements during stops is described in [15]. The cost elements required by the DSF Core are the following:

- *One-off strategy costs:* These costs are defined per machine, component and strategy combination. When multiple strategies start simultaneously to be applied to components of a particular machine, only the maximum one-off strategy cost of the started strategies instead of the sum of all of them is paid and is added to the net costs described later.
- *Net costs:* The stop-type-dependent costs per component are paid during the stop interval. (The DSF Core assumes that they are paid at the beginning of the interval.) They do not include indirect costs, such as cost due to downtime and long-term economic impact, which are taken into account separately.

*Direct impacts of stops on other KPIs:* These are defined by the user and behave like the net costs.

*KPI and failure probability percentiles resolution:* This is defined as *r* as it appears in Algorithm 2.

### 2.2. Scenarios of Running a Trained DSF Core Model

The following two run scenarios have been envisioned:

- *Real-time recommendations scenario:* The trained model runs (near-) real-time (automatically, and periodically, based on the sampling step considered for training) for the next timestamp (based on the sampling step). When a strategy is needed, relevant recommendation is shown. This requires the following real-time input:
  – Stops (production line failures, strategies);
  – Predictions from other DSF algorithms (failure probabilities, degradation levels);
  – Process data related to the KPIs (unless already used directly by other DSF algorithms instead).
- *Simulation scenario:* The trained model runs (manually) for a future time interval, thus simulating (under the trained optimal strategy selection policy and considering the current status of the production line) the following:
  – When–what failures will happen;
  – When–what strategies will be recommended;
  – Future independent KPIs;
  – Future production time percentage.

### 2.3. DSF Core Internal Architecture (Information Flow)

**Figure 1:**
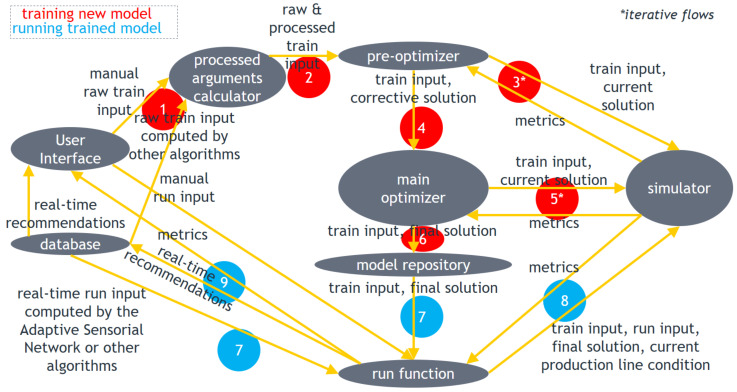
DSF Core architecture. The numbers indicate the sequence of information flows.

The architecture of the DSF Core corresponding to the overall methodology discussed above is outlined in Figure 1. It consists of the following elements:

- *Database:* data repository where sensorial data and outputs of algorithms may be stored for future use by the same or other algorithms within the DSF;
- *User Interface:* helps the end user interact with the DSF Core by inserting inputs and visualizing outputs;
- *Processed arguments calculator:* computes additional training input arguments based on those defined by the user or on output of third-party algorithms;
- *Pre-optimizer:* optimizes the corrective strategies based on Algorithm 1;
- *Main optimizer:* optimizes the preventive strategy application policy based on Algorithm 2;
- *Simulator:* simulates the evolution of the production line condition to evaluate the objective function;
- *Model repository:* working directory where files including the input, processed and trained parameters related to the trained DSF Core models are stored so that the models can run in the future;
- *Run function:* runs trained DSF Core models based on the aforementioned run scenarios.

## 3. Results and Discussion

This section includes the training results from the application of the DSF Core to the three aforementioned pilots, as well as examples of running the respective trained models based on the simulation scenario assuming a new production line in the beginning. For GORENJE and HWH, an indicative DSF Core model is discussed in this paper. For ZORLUTEKS, two representative models are discussed in detail, whereas high-level results for the other trained models for this pilot are also presented in the end.

### 3.1. Application to GORENJE

The spraying cabin is the only machine from the white enameling line of this pilot which is studied by the DSF Core because it involves the most important failures in terms of average yearly impact. The following 48 important components have been selected for analysis:

- 12 power supply units;
- 12 pumps;
- 12 hoses;
- 12 spraying guns.

The components from the different types are in 1-1 correspondence, i.e., there are 12 power supply unit–pump–hose–spraying gun quartets, working in parallel for production.

Due to the high number of components, as well as the assumed independence among components of different quartets in terms of stops, it was decided to train the DSF Core algorithm for only one representative quartet, so that the trained model is used for all of them. Due to the independence of model execution for different quartets, the stop duration corresponding to each quartet is unavoidably charged separately, even in cases when strategies are applied simultaneously to multiple quartets.

#### 3.1.1. Training Input for GORENJE

*Machines of the production line studied by the DSF Core model, components of each machine, as well as potential strategies and failure types corresponding to each component:* According to the above, the model includes one power supply unit, one pump, one hose and one spraying gun. A single failure type has been considered for each such movable component. Maintenance and replacement are the candidate strategies for each component, except for the hose, which may only be replaced.

*Components to be always replaced along with refurbishment:* None.

*Stop types corresponding to decision variables to be excluded from the optimization:* These are the power supply unit and pump strategies, because they are not part of preventive strategies. (The failures of these components are not excluded in order for the DSF Core to select between corrective maintenance and replacement.)

*Lightest compulsory corrective strategies:*

- Power supply unit/pump: maintenance;
- Hose/spraying gun: none.

*Failure type dependencies:* Failures of different types are assumed as independent.

*Sampling step:* 15 min.

*Working hours (from which the gross profit rate is computed):* Working time lasts 8 h per business day (where the working hours have been considered as 9:00–17:00 from Monday to Friday), i.e., 40 h/week, i.e., 2087 h/year (23.81% of total time). Every working hour (woh), 380 parts are produced on average. (This rate corresponds to parts with a width of 50 cm, which is almost always the case. In the following, it will be assumed that all parts have this width). Thus, the ideal production rate is 793,114 parts/year. Under normal conditions, every part is good within the first two processing attempts with 99.6% probability. If it is bad, with the remaining 0.4% probability, it is discarded as scrap. From this percentage and under the assumption that normally each of the two processing attempts has equal probability to produce a good part, it is concluded that this probability is $\sqrt{0.4\%}=6.3\%$. Then, 0.996/(1 + 0.063) = 1 − 0.063 = 93.7% of the produced parts (356 parts/woh) yield profit. The indicative selling price is EUR 7.5/part. Therefore, the estimated ideal gross profit rate is EUR 2669.750/woh = 5,572,150 EUR/year. This is uniformly distributed among all working timestamps based on the relevant sampling step.

*Indication if strategies and failure fixations may take place only during working hours:* Preventive strategies may also take place during non-working time by workers who are in charge during that time; this does not lead to extra labor cost.

*Simulation interval length:* 200 years.

*Remaining equivalent age after strategy compared to before (percentage):*

- Any component—replacement: 0%;
- Pump—maintenance: 10%;
- Spraying gun—maintenance: 5%;
- Power supply unit—maintenance: 20%.

*Load specifications:* The load specifications follow below:

- *Range:* Load is assumed to equal one during production andzero during intervals without production for any analyzed failure type.
- *Maximum absolute first-order difference within 1 day:* Load is sectionally constant;
- *Multiplier during production due to high failure probability until the next timestamp:* One in all cases (no effect assumed);

*KPI types considered:* Economic, scrap.

*Measurement units of KPIs:*

- Economic: Euros;
- Scrap: discarded parts.

*KPI weighting coefficients:* Based on the rate EUR/part during production, the following weighting coefficients of the KPIs are proposed:

- Economic: one;
- Scrap: 7.5.

*Ideal average yearly values of KPIs:*

The production costs of non-degraded equipment follow below:

- Energy: 0.03 kW·2087 woh/year·0.202 EUR/kWh = 12.65 EUR/year = 0.00606 EUR/woh;
- Gas: 0.6 m${}^{3}$/part·793,114 parts/year·1.01 EUR/m${}^{3}$ = 480,627 EUR/year = 230.280 EUR/woh;
- Material: 2.35 kg/part·793,114 parts/year·1.42 EUR/kg = 2,646,621 EUR/year = 1268.06 EUR/woh
- Labor: (as computed in the “cost input” paragraph below) EUR 258,871/year = 124.03125 EUR/woh.

Thus, the production costs of non-degraded equipment are EUR 3,386,132/year = 1622.377 EUR/woh. So, since the average gross profit rate during production is EUR 5,572,150/year = 2669.750 EUR/woh, the average net profit rate would be EUR 2,186,018/year = 1047.374 EUR/woh, i.e., EUR 45,542.05/year = 21.82028 EUR/woh = 5.4551 EUR/(15 womin) for each of the 48 components, if no equipment stop (failure or strategy) occurred (womin = working minutes). (The equipment production efficiency is always assumed as perfect, since no impact of degradation on the KPIs during production has been defined for this pilot.)

Regarding the scrap KPI, as mentioned above, it is known that under normal conditions every part is good within the first two processing attempts with 99.6% probability. If it is bad, with the remaining 0.4% probability, it is discarded as scrap. From this percentage, and under the assumption that normally each of the two processing attempts has equal probability to produce a good part, it is concluded that this probability is $\sqrt{0.4\%}=6.3\%$. Then, 0.004/(1 + 0.063) = 0.38% of the produced parts (1.4 parts/woh = 2984 parts/year) are discarded.

*KPIs evolution and dependencies:* As mentioned above, no impact of degradation on the KPIs during production has been defined for this pilot.

*Stop duration Weibull distribution parameters:*

In contrast to the other DSF Core models, the one-off cost coincides with the duration-dependent cost, as explained later, so the gross duration of simultaneous application of strategies has not been analysed. The gross durations of the individual strategies follow below:

- Replacement of power supply unit: 1 woh;
- Maintenance of power supply unit: 0.3 woh;
- Replacement of pump: 0.45 woh;
- Other: 0.25 woh.

The additional time to fix failures has been defined as follows:

- Power supply unit: negligible, assumed as 0 (because both fixing the failure and maintenance takes about 20 womin);
- Pump: 0;
- Hose: 15 womin = 0.25 woh (for shortening of the hose);
- Spraying gun: 15 womin = 0.25 woh (for disassembly and cleaning of the gun).

The above positive durations are assumed to have coefficient of variation CV = 0.1.

The Weibull distribution parameters are computed from the above as mentioned in the methodology. Particularly, CV = 0.1 corresponds to shape β=12.153434. The scale (η) values for stops of positive duration prove to be as follows:

- Replacement of power supply unit: 1.0430377 woh = 0.043459903 woD (woD = working days);
- Maintenance of power supply unit: 0.31291130 woh = 0.013037971 woD;
- Replacement of pump: 0.46936696 woh = 0.019556957 woD;
- Other strategy/hose failure/spraying gun failure: 0.26075942 woh = 0.010864976 woD.

*ETTF distribution parameters based on equivalent age:* These are shown in Table 3.

*MTTF values for process-data-related component failures, and frequency of their probabilities’ change during production:* Failure occurrences at the components of this machine due to reasons irrelevant to degradation have not been considered.

*Cost input:*

The direct costs during application of strategies are split into duration-dependent (only costs for labor time, since no machines are involved in the application of strategies for this pilot) and duration-independent (costs to buy new components, consumables costs and other costs to apply a strategy which do not depend on its duration). The duration-dependent costs are proportional to the durations of strategies mentioned above. However, usually workers do not work overtime to apply the analysed strategies and are not paid extra for them compared to their fixed reward. The total labor rate is independent of the production and stop time percentages and is defined as 7.5 h/(business day) · 10.5 persons · EUR 12.6/Ph · 5(business days)/week = EUR 4961.25/week = 258,871 EUR/year = 124.03125 EUR/woh (Ph = person hour). During stops, this rate remains constant, but the relevant cost corresponding to the strategy duration is characterized as one-off strategy cost instead. Thus, when multiple strategies are applied simultaneously, the one-off cost corresponding to the strategy that takes longer applies, and the whole duration-dependent cost is considered as one-off. Therefore, the one-off costs, which coincide with the gross duration-dependent costs, are defined as follows:

- Replacement of power supply unit: EUR 124.03125;
- Maintenance of power supply unit: EUR 37.209375;
- Replacement of pump: EUR 55.8140625;
- Other: EUR 31.0078125.

From the above, it also follows that the duration-independent costs coincide with the total net costs.

Table 4 summarizes the direct costs during strategies.

Any failure, depending on its duration, is assumed to have additional cost for labor time (which would be paid also in case of production, as happens with strategies). This means EUR 31.0078125 for a hose and a spraying gun and 0 for the other components. The additional advantage of preventing hose and spraying gun failures is to avoid downtime during working intervals.

*Direct impacts of stops on other KPIs:* When some component fails, this is recognized quickly, so the extra scrap rate applies for a negligible duration. Thus, the extra scrap may be considered as negligible.

*KPI and failure probability percentiles resolution:* 10%.

#### 3.1.2. Training Results for GORENJE

##### Optimal Solution

*Corrective strategies and actuators:* In this case, the initial and corrective solutions coincide. That is, the corrective strategies after failures are the lightest compulsory ones. However, the actuators remained active only for the compulsory maintenance of the power supply unit and the pump after the failure of the corresponding component. A simple fix instead of a strategy is proposed after hose and spraying gun failure.

*Preventive strategies:*

- Preventive replacement is proposed for the hose when its failure probability within the next time step (15 min) exceeds 0.001510.
- Preventive maintenance is proposed for the spraying gun when its failure probability within the next time step (15 min) exceeds 0.001557.

The original training had also proposed preventive maintenance of the power supply unit based on its failure probability, but this was not considered because of the pilot information that preventive maintenance of the power supply unit is impossible.

##### Evaluation Metrics

These are shown in Table 5, Table 6 and Table 7.

#### 3.1.3. Run Results (Simulation Scenario) for GORENJE

**Figure 2:**
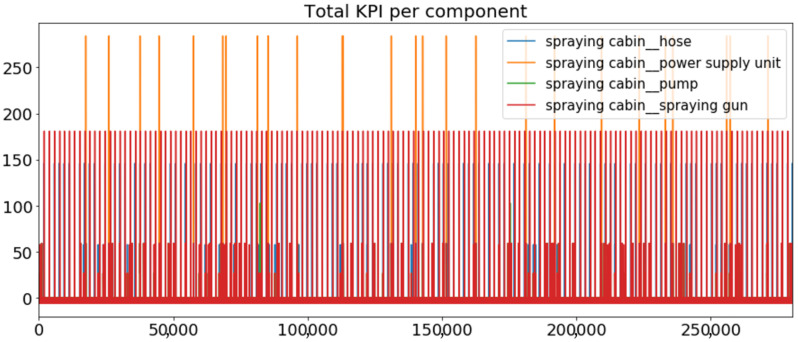
Total KPI time series yielded from running the model for the spraying cabin (GORENJE). The simulation interval has a length of 8 years.

**Figure 3:**
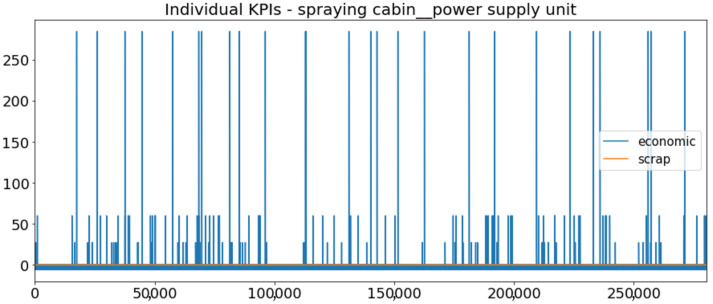
Individual KPI time series for the power supply unit yielded from running the model for the spraying cabin (GORENJE). The simulation interval has a length of 8 years.

**Figure 4:**
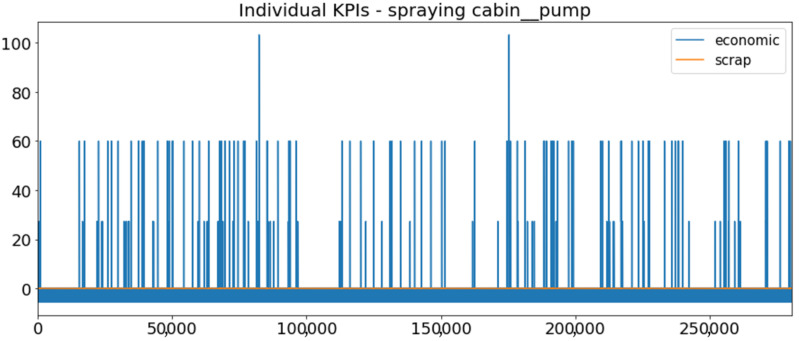
Individual KPI time series for the pump yielded from running the model for the spraying cabin (GORENJE). The simulation interval has a length of 8 years.

**Figure 5:**
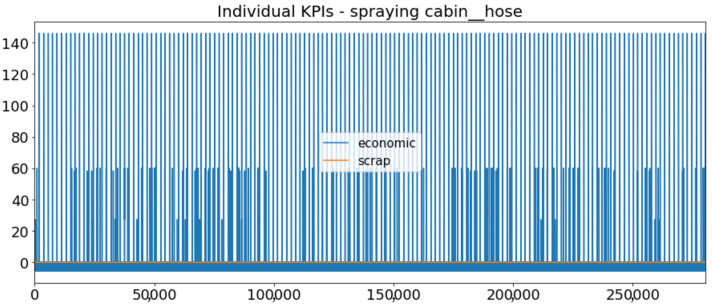
Individual KPI time series for the hose yielded from running the model for the spraying cabin (GORENJE). The simulation interval has a length of 8 years.

**Figure 6:**
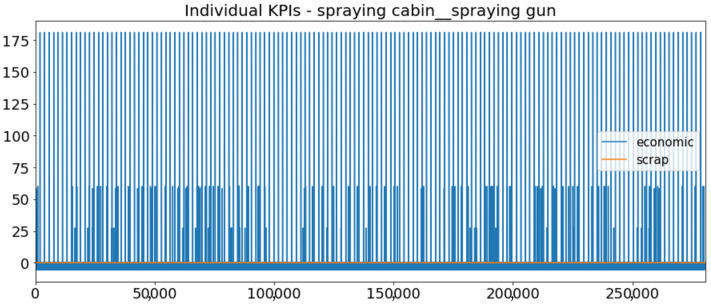
Individual KPI time series for the spraying gun yielded from running the model for the spraying cabin (GORENJE). The simulation interval has a length of 8 years.

Figure 2 presents the time series of the total KPI per component within an interval of 8 years, and the subsequent ones (Figure 3, Figure 4, Figure 5 and Figure 6) show how each KPI is decomposed into individual KPIs. Every perpendicular line corresponds to a stop. The lower-length lines correspond to stop instances of lower impact. When applicable, the downtime impact of stops on the considered components of the production line which are out of this DSF Core model is incorporated in the perpendicular lines. Apparently, during production, the economic and the total KPI per component are negative because overall the production is advantageous. In Figure 2, the values for some components are almost not visible. The first reason for this is that hose replacement and spraying gun maintenance (almost) coincide in time, as also discussed later. Secondly, the pump theoretically fails (and is maintained in a corrective way) only every 4.3 years, based on Table 3, so it is reasonable not to see many pump stops in the 8-year run simulation interval. (A larger simulation interval for the run results was not chosen in this case because stops from other types are very frequent and would hinder the visibility of figures.) Mainly, the downtime impact of stops of other components on the pump is depicted in Figure 4, and this downtime impact coincides in time for all components. As shown in these figures, as well as in Table 5, in this pilot, the total KPI is almost exclusively affected by the economic one.

#### 3.1.4. Discussion of the Training Results for GORENJE

The conclusion from training the model for this pilot is that the lightest possible preventive strategies in the hoses and the spraying guns considerably help to improve the total KPI since they prevent numerous failures and the respective bad consequences. In addition, it was concluded that no corrective strategy should be applied to these components, but the temporary repair solutions should be preferred instead. A hose and a spraying gun have the same lifetime and almost equal failure probability thresholds triggering the preventive strategies on them, and since no corrective strategies are proposed for them, it is preferable to perform preventive strategies for multiple such components simultaneously. In relation to this, it should be noted that the same number of preventive strategies happened in the hose and the spraying gun during the simulation interval under the strategy application policy of the final solution, as shown in Table 6. What is more, regarding the components included in the DSF Core model, preventive replacement of the hose and preventive maintenance of the spraying gun (almost) coincide in time, as shown in Figure 2. Table 8 contains the total KPIs with respect to components obtained from the above trained model and their total in the last row of each sub-table. The scrap penalty is slightly negative (due to downtime), as expected, because no inefficiency during production has been defined for it. Thus, the extra total KPI values, which appear in the bottom right cell of each sub-table and refer to the whole production line, depend almost only on the economic factor. These values indicate that the application of the optimal strategy application policy proposed above may reduce the yearly unnecessary total KPI penalty by 99,467 (40%) compared to the lightest compulsory corrective strategy application policy without preventive strategies. However, as mentioned above, time-based inspection is already taking place at the plant, and based on it, preventive maintenance helps to avoid most failures. In the future, the cost of the time-based inspection policy will be evaluated, thus enabling its comparison with the policy proposed by the DSF Core.

### 3.2. Application to HWH

A friction welding machine from HWH is studied by the DSF.

#### 3.2.1. Training Input for HWH

*Machines of the production line studied by the DSF Core model, components of each machine, as well as potential strategies and failure types corresponding to each component:*Table 9 shows the components of the friction welding machine (one static and four movable), as well as the failure and strategy types considered for each component.

*Components to be always replaced along with refurbishment:* The replacement of the motor and the spindle in case of refurbishment is compulsory.

*Stop types corresponding to decision variables to be excluded from the optimization:* None.

*Lightest compulsory corrective strategies:*

- Static component—failure: refurbishment;
- Motor/spindle—lubricant: none;
- Any other failure type: replacement.

*Failure type dependencies:* Based on prior pilot knowledge, failures of different types are independent, except for the cases of lubricant failure, which immediately cause also mechanical fatigue in the same component.

*Sampling step:* 3 h.

*Working hours (from which the gross profit rate is computed):*

Working time lasts 6 h per business day (Monday-Friday 9:00–15:00), i.e., 30 h/week, i.e., 1565 h/year (17.85% of total time). Each welding action corresponds to one produced part. Welding time lasts 416.67 h/year (0.5 min/part, 50,000 actions/year), so welding time is 416.67/8766 = 4.7533% of total time. Consequently, working time is split into welding time (416.67/1565 = 26.62%) and idle time. Idle time also includes time to repair failures and time to perform strategies, but it is not increased due to these stops, since the lost time is compensated with faster production later. There is no need to produce more than 50,000 parts per year, so this is not calculated, since human work is needed for production. Therefore, the gross profit rate is EUR 500,000/year (from the production of 50,000 parts with a selling price of EUR 10 each). Based on the above, during working time, the average time between welding activities is 0.5·1565/416.67 = 1.878 min (0.5 min welding + 1.378 min idle). The DSF Core does not need such fine time granularity, so welding and idle time will not be distinguished from now on.

During working time, the average gross profit rate from production is EUR 10/ (1.878 min) = 319.4 EUR/h. This verifies that the average gross profit rate is EUR 500,000/year (EUR 10 · 50,000 welding activities).

*Indication if strategies and failure fixations may take place only during working hours:* The restoration of production may also be completed during non-working intervals.

*Simulation interval length:* 292 years.

*Remaining equivalent age after strategy compared to before (percentage):*

- Static component—refurbishment—failure: 0%;
- Motor—maintenance:
  – Mechanical fatigue: 100%;
  – Lubricant: 0%.
- Motor—replacement:
  – Mechanical fatigue: 0%;
  – Lubricant: 0%.
- Spindle—maintenance:
  – Mechanical fatigue: 100%;
  – Lubricant: 0%.
- Spindle—replacement:
  – Mechanical fatigue: 0%;
  – Lubricant: 0%.

*Load specifications:* The load specifications follow below:

- *Range:* In most cases, it is assumed that normal usage corresponds to average load of 1, ranging from 0.5 to 1.5, as it depends on the way the equipment is used, expressed by potential sensorial data relevant to load calculation throughout working (instead of welding) time. Exceptionally, the load during working time for the static component is assumed by the pilot to range from 0.9 to 1.1. Furthermore, for the sample-detector failure type, load is assumed to exactly equal one during working time (and zero otherwise) only for the purpose of economic penalty evaluation as a function of equivalent age, because the sample-detector failures may be better forecast based on process data anomalies rather than equivalent age. In addition, for the lubricant failure type of the motor and the spindle, load is assumed to exactly equal one during the whole time, including non-working intervals.
- *Maximum absolute first-order difference within 1 day:* 0.72.
- *Multiplier during production due to high failure probability until the next timestamp:* one in all cases (no effect assumed).

*KPI types considered:* economic.

*Measurement units of KPIs:* Euros.

*KPI weighting coefficients:* one.

*Ideal average yearly values of KPIs:* The production costs of non-degraded equipment follow below:

- Energy: EUR 520/year = 0.33 EUR/woh;
- Gas: 0.025 m${}^{3}$/part·50,000 parts/year· EUR 1.6/kg·1.225 kg/m${}^{3}$ = 2450 EUR/year = 1.565 EUR/woh;
- Labor: 0.05 min/part·50,000 parts/year· EUR 27.4/h = 1141.67 EUR/year = 0.7293 EUR/woh;
- Pressurized air: EUR 220/year = 0.14 EUR/woh;
- Cooling water: EUR 200/year = 0.13 EUR/woh.

Thus, the production costs of non-degraded equipment are EUR 4531.67/year. Therefore, since the average gross profit rate is EUR 500,000/year, the average net profit rate would be EUR 495,468.33/year, i.e., EUR 63.3/woh = 189.9 EUR/(3 woh) for each of the five components, if no equipment stop (failure or strategy) occurred, and the equipment production efficiency was always perfect.

*KPIs evolution and dependencies:* The extra production costs due to degradation (supposedly at 0.9 METTF${}_{c}$, where METTF${}_{c}$ is the METTF of component *c* based on equivalent age) are caused by extra power of 0.01 kW, i.e., 0.002 kW = 0.048 kWh/(working day) for each of the five components. During working time, this costs 0.048 kWh/D·0.14 EUR/kWh = 0.00672 EUR/D per component.

*Stop duration Weibull distribution parameters:*

The gross durations of strategy combinations are:

1. Refurbishment of static component (excluding the compulsory simultaneous replacement of the motor and the spindle): TTR_static,refurbishment_ + TTR_fwm_ = 16 woh;
2. Replacement of motor: TTR_motor,replacement_ + TTR_fwm_ = 8 woh;
3. Replacement of spindle: TTR_motor,replacement_ + TTR_fwm_ = 8 woh;
4. Replacement of sample-holder: TTR_sh,replacement_ + TTR_fwm_ = 1 woh;
5. Replacement of sample-detector: TTR_sd,replacement_ + TTR_fwm_ = 1 woh;
6. Refurbishment of static components and replacement of all movable components: TTR_static,refurbishment_ + TTR_motor,replacement_ + TTR_spindle,replacement_ + TTR_sh,replacement_ + TTR_sd,replacement_ + TTR_fwm_ = 20 woh.

TTR_fwm_ stands for the one-off duration required during every group of simultaneous strategies applied to the friction welding machine.

Solving the above system of six equations with six unknown variables with respect to these variables (independent net durations and TTR_fwm_) yields negative values for TTR_sh,replacement_ and TTR_sd,replacement_, so this solution should be rejected. To face this issue, TTR_fwm_ is removed from the fourth and fifth problematic equations, which yields the following acceptable solution:

- One-off duration: TTR_fwm_ = 7 woh;
- Refurbishment of static component (excluding the compulsory simultaneous replacement of the motor and the spindle): TTR_static,refurbishment_ = 9 woh;
- Replacement of any movable component: TTR_motor,replacement_ = TTR_spindle,replacement_ = TTR_sh,replacement_ = TTR_sd,replacement_ = 1 woh.

Thus, it seems that the one-off duration appears if and only if at least one component among the static ones, the motor and the spindle undergo some strategy, i.e., it does not apply when only the sample-holder and/or the sample-detector is replaced. Therefore, the gross duration of refurbishment, including the compulsory simultaneous replacement of the motor and the spindle, is 18 woh.

In addition, it has been assumed that maintenance of motor and spindle has the same duration as replacement of these components, respectively.

Based on the above, the downtime does not cause indirect cost due to lost production profit because the lost time is compensated. Thus, no downtime is modelled. The durations were computed only to evaluate the costs of strategies on the movable components, including the duration-dependent costs, which have been explicitly provided by the pilot only for refurbishment.

Since the lost time is compensated, the duration of failures has not been modelled. No duration-dependent failure costs have been analyzed.

To conclude, the duration of every stop from the downtime viewpoint has been assumed as 0.

*ETTF distribution parameters based on equivalent age:* These are shown in Table 10.

*MTTF values for process-data-related component failures, and frequency of their probabilities’ change during production:* The relevant failure is the remaining sample-holder failure. The MTTF related to process-data-related reasons for this failure type has been assumed as 1.5Γ(1+1/2) years = 1.33 years = 86.68 working days = 553.89 weh (weh = welding hours). Apart from the cases of strategies, the probability obtained from the above distribution changes with a new random value every 5 woDon average.

*Cost input:*

The direct costs during application of strategies are split into duration-dependent (costs for machine time and labor time) and duration-independent (costs to buy new components, consumables costs and other costs to apply a strategy which do not depend on its duration). Since the machine time always equals the labor time in the case of this pilot, the duration-dependent costs are proportional to the durations of strategies mentioned above, according to the sum of the defined labor rate (EUR 27.4/h) and machine rate (0.5 EUR/h), so EUR 27.9/h. The one-off cost, corresponding to the one-off duration, is EUR 195.3, so this is the difference between gross and net costs. This is paid only once for every group of strategies applied simultaneously to the machine, unless only the sample-holder and/or sample detector are/is involved, where this cost is not paid.

Table 11 summarizes the direct costs during strategies.

Fixing of any failure without application of some strategy is assumed to have a direct cost of EUR 2000.

*Direct impacts of stops on other KPIs:* No other KPIs have been considered for this model.

*KPI and failure probability percentiles resolution:* 5% (10% proved to be too high).

#### 3.2.2. Training Results for HWH

##### Optimal Solution

*Corrective strategies and actuators:* In this case, the initial and corrective solution coincide. That is, the corrective strategies after failures are the lightest compulsory ones. However, the actuators remained active only for the compulsory strategies after failures. A simple fixing instead of a strategy is proposed after motor and spindle lubricant failure, which is reasonable because mechanical fatigue always follows lubricant failure, which necessitates replacement of the component anyway.

*Preventive strategies:*

- Preventive maintenance is proposed for the:
  – Motor when:
    * The short-interval average modified KPI for the maintenance of this component exceeds $6.750156\cdot 10^{-14}$;
    * Its lubricant failure probability within the next time step (3 h) exceeds $1.13\cdot 10^{-4}$.
  – Spindle when the short-interval average modified KPI for the maintenance of this component exceeds $5.684342\cdot 10^{-14}$.
- Preventive replacement is proposed for the
  – Sample-holder when its failure probability within the next time step (3 h) exceeds $3.44\cdot 10^{-4}$;
  – Sample-detector when its failure probability within the next time step (3 h) exceeds $2.38\cdot 10^{-4}$.

##### Evaluation Metrics

These are shown in Table 12 and Table 13.

Apparently, since no downtime has been modelled for this pilot, the production time percentage for every component equals the ideal (17.8572%) in every case.

#### 3.2.3. Run Results (Simulation Scenario) for HWH

**Figure 7:**
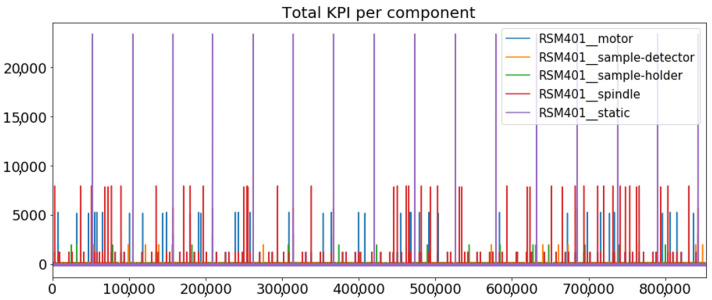
KPI time series for every component yielded from running the model for the friction welding machine (HWH). The simulation interval has the same length as for training (292 years).

**Figure 8:**
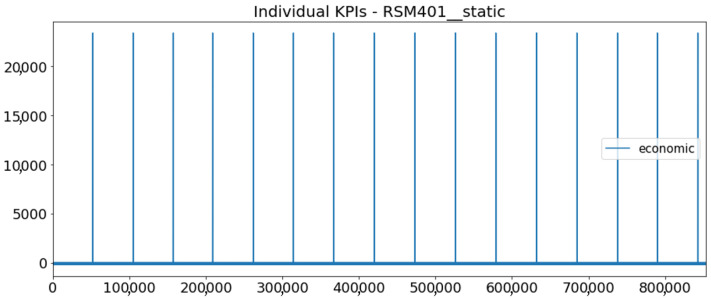
KPI time series for the static component yielded from running the model for the friction welding machine (HWH). The simulation interval has the same length as for training (292 years).

**Figure 9:**
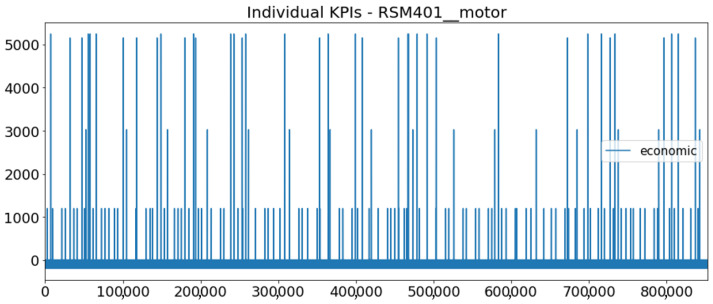
KPI time series for the motor yielded from running the model for the friction welding machine (HWH). The simulation interval has the same length as for training (292 years).

**Figure 10:**
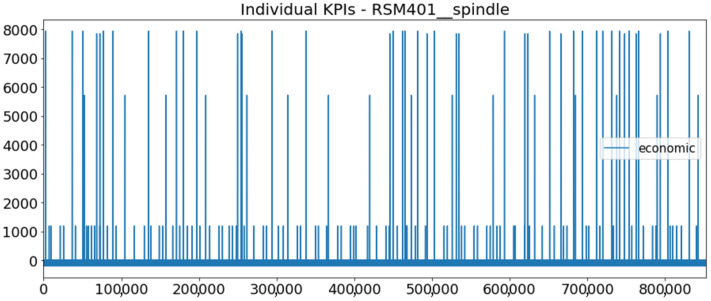
KPI time series for the spindle yielded from running the model for the friction welding machine (HWH). The simulation interval has the same length as for training (292 years).

**Figure 11:**
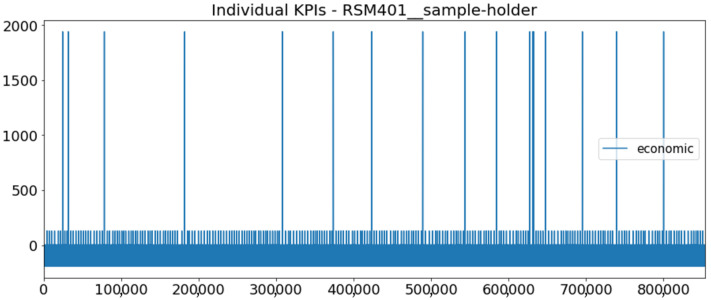
KPI time series for the sample-holder yielded from running the model for the friction welding machine (HWH). The simulation interval has the same length as for training (292 years).

**Figure 12:**
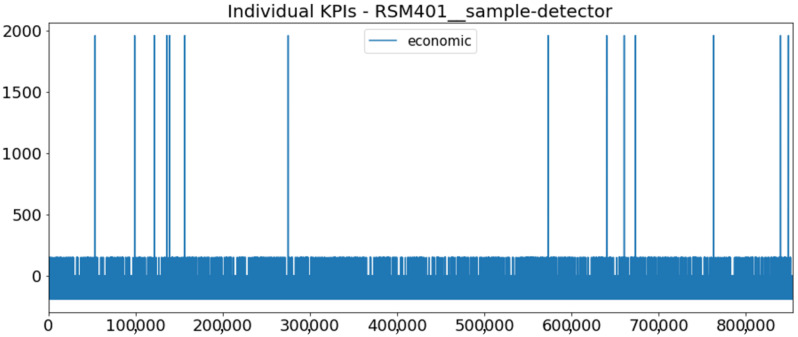
KPI time series for the sample-detector yielded from running the model for the friction welding machine (HWH). The simulation interval has the same length as for training (292 years).

Figure 7 presents the time series of the total KPI (which coincides with the economic KPI, since no other KPIs have been considered for this pilot) per component within an interval of the same length as the simulation interval during training, and the subsequent ones (Figure 8, Figure 9, Figure 10, Figure 11 and Figure 12) show this KPI for each component separately. Every perpendicular line corresponds to a stop. The lower-length lines correspond to stop instances of lower impact.

#### 3.2.4. Discussion of the Training Results for HWH

The conclusion from training the above model for this pilot is that the lightest possible preventive strategies in the movable components considerably help to improve the KPI, since they prevent numerous failures and the respective bad consequences. Based on the simulations, the number of replacement strategies in the sample-detector under the optimal strategy application policy is much higher than the number of replacement strategies in the sample-holder. This happens not only because the sample-detector has about half lifetime, but also because the sample-detector failure probabilities have been modelled as process-data-related instead of equivalent-age-based (which is the case for the other components), and as a result, the prevention thanks to the strategy has more short-term effect; 5 working days on average, based on the input.

The results indicate that the application of the optimal strategy application policy proposed above may reduce the yearly unnecessary total KPI penalty by EUR 2168/year (27%) compared to the lightest compulsory corrective strategy application policy without preventive strategies.

### 3.3. Application to ZORLUTEKS

The bleaching machine of the production line is studied in this work. The following 51 important components have been selected for analysis:

- 14 roller coatings (one for each of the 16 analysed rollers except the 2 standalone—the other 14 coated rollers are in pairs);
- 16 double bearing-lubricant pairs (one for each roller);
- 10 inverters;
- 11 motors.

Due to the high number of components and the similarity among many of them, it was decided to train the DSF Core algorithm separately for different components, except for those with correlated failures and load. While strategies are applied simultaneously to components which belong to the same DSF Core model, the corresponding duration and the one-off strategy cost related to the waiting time to insert fabric into the machine are considered only once. In all other cases, due to the independence of the models, unavoidably the duration corresponding to each component and the one-off strategy cost are charged separately.

To keep its content concise, this paper will only discuss two indicative DSF Core models for ZORLUTEKS. The first is related with one of the seven motors with two associated paired coated rollers each. The other models for motors with two rollers are not presented in detail, since they only differ in the METTF of some components. The second model for this pilot discussed in this paper is related with any of the two motors with one associated roller each. These two rollers are the uncoated ones. The model for motors without associated rollers and the models for inverters are also not discussed in detail because they are simpler, and in these cases the benefit from applying preventive strategies is less significant.

#### 3.3.1. Training Input for ZORLUTEKS

*Machines of the production line studied by the DSF Core model, components of each machine, as well as potential strategies and failure types corresponding to each component:*

Table 14 shows the types of movable components of the bleaching machine considered by the two DSF Core models, as well as the failure and strategy types considered for each component. Based on the above, the first model considers five components; two roller coatings, two double bearing-lubricant pairs (each corresponding to one roller) and one motor connected to the two rollers. The second model considers two components; one double bearing-lubricant pair of a uncoated roller and one associated motor.

Although there are several other stop types, they have not been modelled because they have been considered as non-controllable or of insignificant impact on the KPIs, and according to a preliminary statistical stop correlation analysis based on [78], there was no (reliable) conclusion about their dependence with the important failure types mentioned above. Thus, for simulation purposes, the production time is uniformly distributed outside intervals of important failures and application of strategies. (As discussed later, all hours of the week are considered as working hours.)

*Components to be always replaced along with refurbishment:* None.

*Stop types corresponding to decision variables to be excluded from the optimization:* None.

*Lightest compulsory corrective strategies:* maintenance of the failed component (winding for complete motor failure, and lubrication for motor lubricant failure).

*Failure type dependencies:* Based on prior knowledge of the domain experts, any bearing lubricant failure immediately causes also mechanical fatigue in the bearing-lubricant pair. There are also load-related dependencies, as explained later.

*Sampling step:* 30 min.

*Working hours (from which the gross profit rate is computed):*

Based on the cost modelling template, about 3 Mm${}^{2}$ of fabric are produced per month. More accurately, based on a preliminary analysis of a production dataset from 2020 and 2021, the time and average yearly output area of fabric are distributed as shown in Table 15.

The bleaching route operation is the only one considered as related to profit. Its time percentage may be improved thanks to third quality optimization algorithms with respect to process parameters by up to 7.55 percentage units (from 54.28% to 61.83%), i.e., the percentage of the bleaching repair operation timestamps, which can be avoided thanks to improvement of the initial quality of products (i.e., after the first process and before any potential reprocess). Since, based on the above table, the average bleaching speed during route operation is 27.72 Mm${}^{2}$/year, there is potential to increase the yearly production rate of profitable bleached fabric to 31.58 Mm${}^{2}$/year (33.55 Mm${}^{2}$/year including washing), which corresponds to gross profit rate of EUR 25.90 M/year, since the unit gross profit is 0.82 EUR/m${}^{2}$. No fixed non-working hours within the week exist, and the DSF Core is generally impossible to know a priori the intervals without bleaching route operation, so, for the simulations, it uniformly distributes the profitable production rate (considering gross profit) as 60.04 m${}^{2}$/min (63.79 m${}^{2}$/min including washing) = 49.23 EUR/min = 2954 EUR/h. For the purpose of evaluation of the economic KPI per component, it is assumed that each of the 51 components of the machine contributes equally to the gross profit rate by EUR 2954/h/51 = 57.92 EUR/h.

*Indication if strategies and failure fixations may take place only during working hours:* non-applicable (all hours are working hours).

*Simulation interval length:* 200 years.

*Remaining equivalent age after strategy compared to before (percentage):*

- Any component—replacement: 0% for each failure type;
- Roller coating—maintenance: 10%;
- Double bearing-lubricant pair—maintenance:
  – Bearing mechanical fatigue: 100%;
  – Bearing lubricant: 0%.
- Motor—maintenance (lubrication):
  – Complete: 100%;
  – Lubricant: 0%.
- Motor—maintenance (winding):
  – Complete: 10%;
  – Lubricant: 100%.

**Figure 13:**
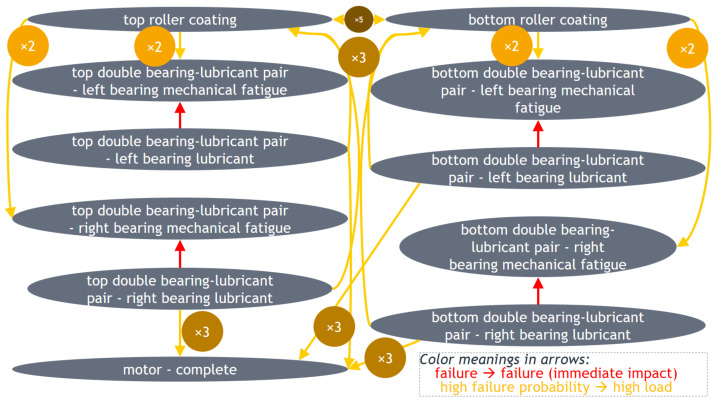
Failure-type dependencies and load multipliers for the bleaching machine (ZORLUTEKS).

*Load specifications:* The load specifications follow below:

- *Range:* Normally, load is assumed to equal one during production and zero during intervals without production for any mechanical fatigue of bearing and complete motor failure. For the other failure types, load is assumed asone all the time. The only exceptions which apply are related to the multipliers discussed below.
- *Maximum absolute first-order difference within 1 day:* load is sectionally constant.
- *Multiplier during production due to high failure probability until the next timestamp:* According to the orange arrows in Figure 13, load with respect to a particular failure type (arrow end) is assumed to be multiplied by the respective number when the probability of failure of another type (arrow start) within the next elementary time interval (i.e., interval with length equal to the sampling step) is relatively high (i.e., higher than the theoretical degradation-based probability corresponding to 90% of METTF). The red arrows indicate the aforementioned failure correlations.

*KPI types considered:* economic, energy, other environmental (water, steam, material)

*Measurement units of KPIs:*

- economic: EUR.
- energy: kWh.
- other environmental: kg.

*Ideal average yearly values of KPIs:*

The production costs of non-degraded equipment follow below:

- Water: 0.0023 m${}^{3}$/(m${}^{2}$ of fabric) · 33.55 (Mm${}^{2}$ of fabric)/year · 0.165 EUR/m${}^{3}$ = 12,732 EUR/year = 1.4525 EUR/h;
- Energy: 0.0051 kWh/(m${}^{2}$ of fabric) · 33.55 (Mm${}^{2}$ of fabric)/year · 0.07 EUR/kWh = 11,977 EUR/year = 1.3663 EUR/h;
- Steam: 0.41 kg/(m${}^{2}$ of fabric) · 33.55 (Mm${}^{2}$ of fabric)/year · 0.012EUR/kg = 165,066 EUR/year = 18.8303 EUR/h
- Material (fabric): 31.58 (Mm${}^{2}$ of fabric)/year · 0.48 EUR/(m${}^{2}$ of fabric) = 15,158,400 EUR/year = 1729 EUR/h;
- Labor: 225 Phs/month·2.3 EUR/Ph = 517.5 EUR/month = 6210 EUR/year = 0.7084189 EUR/h (may be considered as negligible compared to the other production costs—paid also during stops).

Therefore, production costs of non-degraded equipment are EUR 15.35 M/year = 1751 EUR/h. Thus, since the average gross profit rate is EUR 25.90 M/year, the average net profit rate would be EUR 10.55 M/year = 1203 EUR/h, i.e., EUR 206,863/year = 23.58 EUR/h = 11.79 EUR/(30 min) for each of the 51 components if no equipment stop (failure or strategy) or any bleaching repair operation occurred. (The equipment production efficiency is always assumed as perfect, since no impact of degradation on the KPIs during production has been defined for this pilot.)

Accordingly, the environmental impact of non-degraded equipment due to production is:

- Energy: 0.0051 kWh/(m${}^{2}$ of fabric) · 33.55 (Mm${}^{2}$ of fabric)/year = 171,105 kWh/year = 19.5192 kW → 3355 kWh/year = 0.382729 kW = 0.191365 kWh/(30 min) for each of the 51 components;
- Other environmental (total resulting from the following): 14,084.3 t/year = 1606.70 kg/h → 276,163 kg/year = 31.5039 kg/h = 15.7520 kg/(30 min) for each of the 51 components:
  – Water: 0.0023 m${}^{3}$/(m${}^{2}$ of fabric) · 33.55 (Mm${}^{2}$ of fabric)/year = 77,165 m${}^{3}$/year = 77,165 kg/year;
  – Chemicals: 0.0075 kg/(m${}^{2}$ of fabric) · 33.55 (Mm${}^{2}$ of fabric)/year = 251,625 kg/year;
  – Steam: 0.41 kg/(m${}^{2}$ of fabric) · 33.55 (Mm${}^{2}$ of fabric)/year = 13,755.5 t/year.

*KPI weighting coefficients:* Based on the average yearly rates EUR/kWh and EUR/kg during production obtained from the above, the following weighting coefficients of the KPIs are proposed:

- Economic: 1;
- Energy: 0.07;
- Other environmental: (12,732 + 124,741 + 165,066)/(77,165 + 251,625 + 13,755,500) = 0.021481.

*KPIs evolution and dependencies:* As mentioned above, no impact of degradation on the KPIs during production has been defined for this pilot.

*Stop duration Weibull distribution parameters:*

The gross durations of strategies (replacement/maintenance) for the involved components are:

- Roller coating: 435 min = 7.25 h;
- Double bearing-lubricant pair: 80 min = 1.33…h (the maintenance is applied in parallel to the production);
- Motor: 210 min = 3.5 h (except for lubrication, for which the duration is 20 min = 0.33…h—the lubrication is applied in parallel to the production).

Unless stated that a strategy is applied in parallel to the production, in which case the strategy duration is considered as 0 in the sense that it does not imply downtime, the fabric should have been removed from the machine so that it takes place. This results in additional waiting time of 23 min, which is the time needed for the fabric to go through the machine.

The above durations are assumed to have coefficient of variation CV = 0.1.

The Weibull distribution parameters are computed from the above as mentioned in the methodology. Particularly, CV = 0.1 corresponds to shape β=12.153434. The scale (η) values for strategies not applied in parallel to the production prove to be as follows:

- Replacement/maintenance of roller coating: 7.5620232 woh = 0.31508430 woD;
- Replacement of double bearing-lubricant pair: 1.3907169 woh = 0.057946538 woD;
- Replacement/maintenance (winding) of motor: 3.6506319 woh = 0.15210967 woD.

Based on the analysis of stop data from 2016 to 2021 for the mechanical, electrical and power failures, the mean and CV statistics were computed for the involved component types. Based on them, the most appropriate scale and shape of two-parameter Weibull distribution parameters were computed, as shown in Table 16, so these distributions are used for the simulation of failure intervals for the evaluation of the objective function of the optimization algorithm.

As discussed below more extensively, each failure causes bad fabric whiteness with a probability dependent on its type. If bad whiteness is detected at the exit of the production line, the same fabric is re-inserted into the machine and reprocessed, which is supposed to require another 23 min. However, when the failure happens, the fabric does not need to be removed from the machine to fix the failure. This may be required only by the strategy applied after the failure, according to the above.

*ETTF distribution parameters based on equivalent age:* These are shown in Table 17.

*MTTF values for process-data-related component failures and frequency of their probabilities’ change during production:* Failure occurrences at the components of this machine due to reasons irrelevant to degradation have not been considered. As mentioned above, it is assumed that lubricant failure in a particular bearing-lubricant pair may be well-forecast thanks to vibration anomalies detection, but this is already considered by the respective modified degradation model, since it is considered as degradation-related.

*Cost input:*

No costs for machine time and consumables costs have been defined for this pilot, and the costs for labor time should not be considered, because the total payment for the staff is constant. Thus, all in all, there are no direct duration-dependent costs during application of strategies, and all direct costs are duration-independent (costs to buy new components and other costs to apply a strategy which do not depend on its duration). The one-off cost, corresponding to the waiting time to insert fabric into the machine after its removal for the purpose of application of a strategy, leads to economic impact equal to the respective gross profit loss, i.e., 23 min · EUR 49.23/min = EUR 1132, i.e., EUR 22.20 for each of the 51 components. This is the difference between gross and net costs when applicable, i.e., for strategies for which the removal of the fabric is necessary.

Table 18 summarizes the direct costs during strategies for the involved components.

On average, any failure, no matter the strategy to be applied afterwards, is assumed to have additional cost because it involves a probability that whiteness becomes bad, defined by the pilot experts as shown in Table 19, in which case the fabric needs to be reprocessed. Thus, the average gross profit lost during the waiting time for the fabric of bad whiteness to exit the machine (assumed as 23 min) should be considered extra. The aforementioned bad whiteness probabilities should be multiplied by this duration to compute probabilistically the expected additional waiting time shown in the column “Expected waiting time due to bad whiteness” of the table and the respective additional cost based on the rate EUR 49.23/min.

The additional advantages of preventing failures is to avoid the downtime during the failure and the negative impact of anomalies and failures on the lifetime of neighboring components, according to the above.

*Direct impacts of stops on other KPIs:*

The assumptions about the average waste per discarded component follow below:

- Roller coating: 36 kg;
- Double bearing-lubricant pair: 13 kg;
- Motor: 100 kg (lubricant: 0.1 kg).

This waste is assumed to appear when the respective component is replaced. (Motor lubricant waste corresponds to motor lubrication.)

*KPI and failure probability percentiles resolution:* 10%.

#### 3.3.2. Training Results for ZORLUTEKS—Model with Paired Coated Rollers

##### Optimal Solution

Initially, the optimal strategy application policy for the double bearing-lubricant pair of the bottom roller was different from the policy for that of the top one, but the latter proved to be better and more stable across simulations, so it was selected for both double bearing-lubricant pairs.

*Corrective strategies and actuators:* The corrective strategies are mentioned in Table 20. All actuators are active because there is always some compulsory strategy after any failure, according to the above.

*Preventive strategies:* Preventive maintenance is proposed for the

- Coating of the bottom roller when its failure probability within the next time step (30 min) exceeds $4.6\cdot 10^{-5}$;
- Coating of the top roller when its failure probability within the next time step (30 min) exceeds $4.4\cdot 10^{-5}$ (close to the case of the coating of the bottom roller);
- Double bearing-lubricant pair of any roller when the short-interval average modified KPI for the maintenance of this component exceeds −5.723695048214355;
- Motor (lubrication) when the short-interval average modified KPI for the maintenance (lubrication) of this component exceeds −5.723695048214355.

Although the increase of KPI inefficiency due to equivalent aging has been defined as negligible for this pilot, KPI thresholds are still proposed, as shown above. Thus, the effective run of the model without surprising results requires precise evaluation of KPIs and definition of their thresholds. Otherwise, the optimal policy found after training the model with standalone uncoated rollers (Section 3.3.4) should be considered instead for the double bearing-lubricant pairs and the motor, because it depends only on failure probabilities (which increase much faster in absolute values due to degradation) and not on KPIs.

##### Evaluation Metrics

These are shown in Table 21, Table 22 and Table 23.

#### 3.3.3. Run Results (Simulation Scenario) for ZORLUTEKS—Model with Paired Coated Rollers

**Figure 14:**
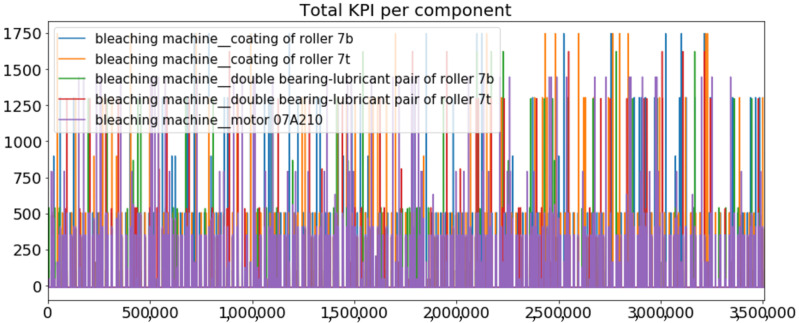
Total KPI time series yielded from running the model with paired coated rollers for the bleaching machine (ZORLUTEKS). The simulation interval has the same length as for training (200 years).

**Figure 15:**
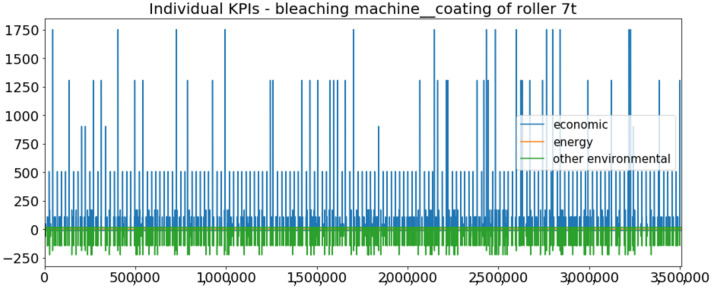
Individual KPI time series for the coating of the top roller yielded from running the model with paired coated rollers for the bleaching machine (ZORLUTEKS). The simulation interval has the same length as for training (200 years).

**Figure 16:**
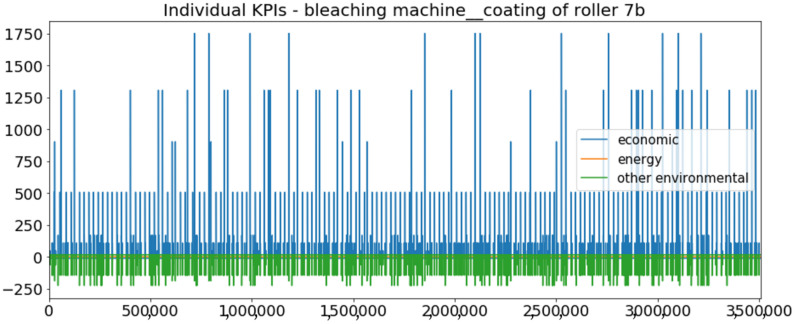
Individual KPI time series for the coating of the bottom roller yielded from running the model with paired coated rollers for the bleaching machine (ZORLUTEKS). The simulation interval has the same length as for training (200 years).

**Figure 17:**
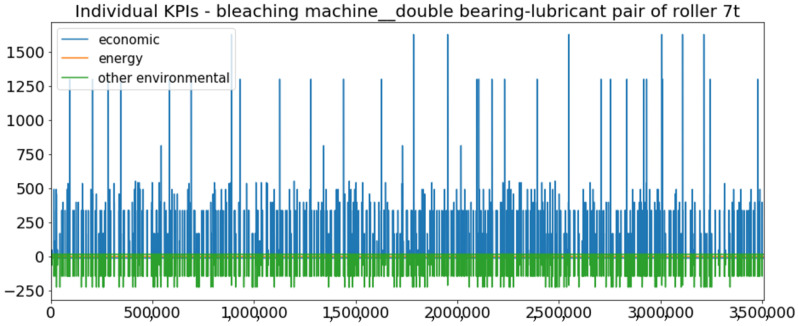
Individual KPI time series for the double bearing-lubricant pair of the top roller yielded from running the model with paired coated rollers for the bleaching machine (ZORLUTEKS). The simulation interval has the same length as for training (200 years).

**Figure 18:**
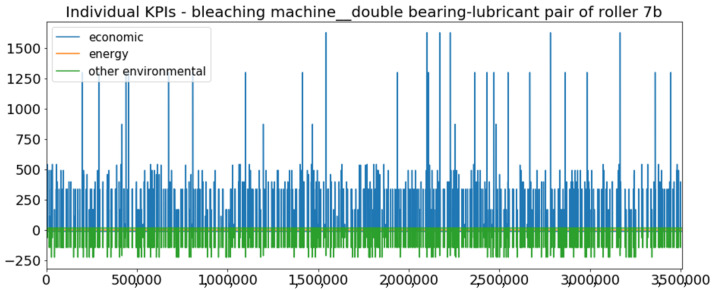
Individual KPI time series for the double bearing-lubricant pair of the bottom roller yielded from running the model with paired coated rollers for the bleaching machine (ZORLUTEKS). The simulation interval has the same length as for training (200 years).

**Figure 19:**
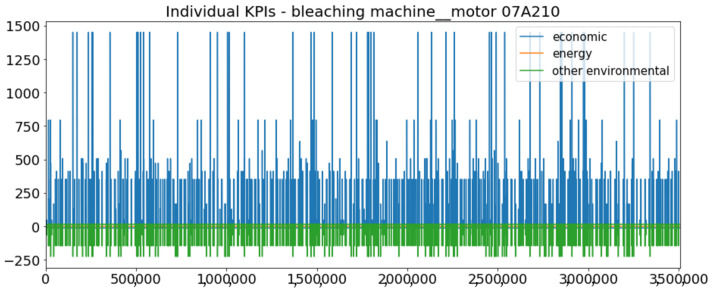
Individual KPI time series for the motor yielded from running the model with paired coated rollers for the bleaching machine (ZORLUTEKS). The simulation interval has the same length as for training (200 years).

Figure 14 presents the time series of the total KPI per component within an interval of same length as the simulation interval during training, and the subsequent ones (Figure 15, Figure 16, Figure 17, Figure 18 and Figure 19) show how each KPI is decomposed into individual KPIs. Every perpendicular line corresponds to a stop. The lower-length lines correspond to stop instances of lower impact. When applicable, the downtime impact of stops on the considered components of the production line which are out of this DSF Core model is incorporated in the perpendicular lines, which explains the negative values of the other environmental KPI.

#### 3.3.4. Training Results for ZORLUTEKS—Model with Standalone Uncoated Roller

##### Optimal Solution

The solution below, which was found from the original training, proved to be comparable with the solution based on the optimal strategy application policy for double bearing-lubricant pairs and motors as selected for models for motors with two rollers.

*Corrective strategies and actuators:* The corrective strategies are mentioned in Table 24. All actuators are active because there is always some compulsory strategy after any failure, based on the above.

*Preventive strategies:* Preventive maintenance is proposed for the

- double bearing-lubricant pair when:
  – Its left bearing lubricant failure probability within the next time step (30 min) exceeds $9.113753\cdot 10^{-9}$;
  – Its right bearing lubricant failure probability within the next time step (30 min) exceeds $3.796748\cdot 10^{-8}$.
- Motor when its lubricant failure probability within the next time step (30 min) exceeds $3.840225\cdot 10^{-5}$ (in which case the maintenance is lubrication).

##### Evaluation Metrics

These are shown in Table 25, Table 26 and Table 27.

#### 3.3.5. Run Results (Simulation Scenario) for ZORLUTEKS—Model with Standalone Uncoated Roller

**Figure 20:**
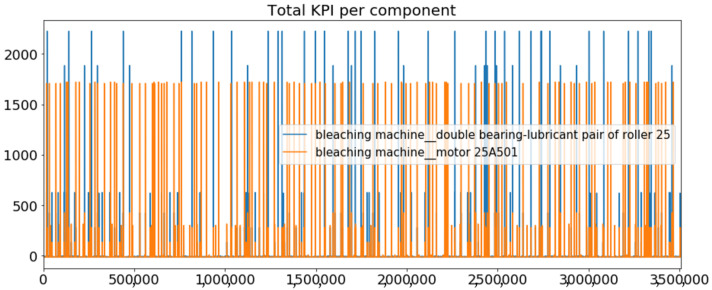
Total KPI time series yielded from running the model with standalone uncoated roller for the bleaching machine (ZORLUTEKS). The simulation interval has the same length as for training (200 years).

**Figure 21:**
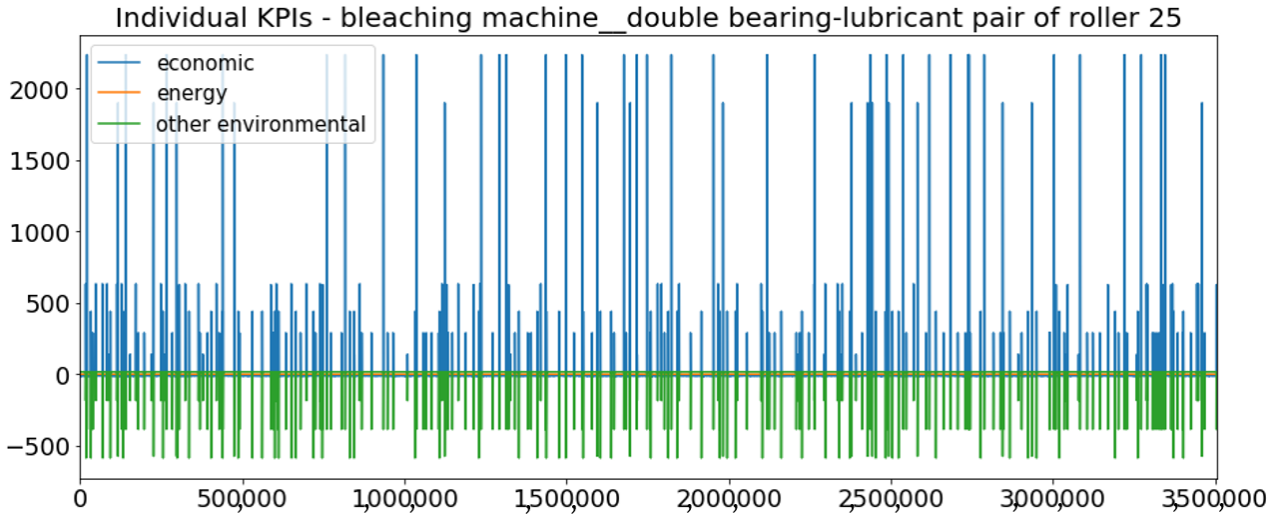
Individual KPI time series for the double bearing-lubricant pair yielded from running the model with standalone uncoated roller for the bleaching machine (ZORLUTEKS). The simulation interval has the same length as for training (200 years).

**Figure 22:**
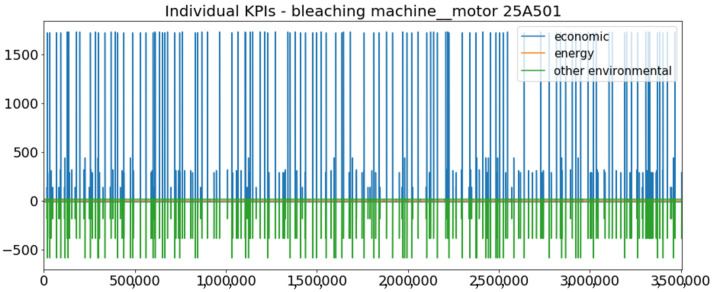
Individual KPI time series for the motor yielded from running the model with standalone uncoated roller for the bleaching machine (ZORLUTEKS). The simulation interval has the same length as for training (200 years).

Similar to before, Figure 20 presents the time series of the total KPI per component within an interval of the same length as the simulation interval during training, and the subsequent ones (Figure 21 and Figure 22) show how each KPI is decomposed into individual KPIs. Every perpendicular line corresponds to a stop. The lower-length lines correspond to stop instances of lower impact. When applicable, the downtime impact of stops on the considered components of the production line which are out of this DSF Core model is incorporated in the perpendicular lines, which explains the negative values of the other environmental KPI.

#### 3.3.6. Discussion of the Training Results for ZORLUTEKS

The conclusion from training the above models for this pilot is that the fast and economic preventive maintenance strategies, which usually may happen in parallel to production, help to considerably improve the total KPI, since they prevent numerous failures and the respective bad consequences. In addition, the fact that replacement instead of maintenance is preferred for a double bearing-lubricant pair after bearing mechanical fatigue for one of its bearings is because maintenance does not decrease at all the equivalent age with respect to mechanical fatigue. Table 28 contains the total KPIs with respect to components obtained from all seven trained models and their sum in the last row of each sub-table. (As mentioned above, the details about the extra five models are omitted in this paper to avoid extending its content too much.) As shown in the table, all but the last model correspond to solutions better than the respective initial ones; thus these models improve the total KPI. The models discussed above in detail correspond to the names “Motor of type 2_3_7” (for paired coated rollers) and “Motor of type 1” (for a standalone uncoated roller). Similar to GORENJE, the multipliers in the second column indicate the number of identical (groups of) components, to all of which the respective models apply. The energy and other environmental penalties are slightly negative (due to downtime), as expected, because no inefficiency during production has been defined for them, whereas the only defined non-economic penalty during stops is waste, which is almost negligible for the selected KPI weighting. Thus, the extra total KPI values, which appear in the bottom right cell of each sub-table and refer to the whole production line, depend almost only on the economic factor. These values indicate that the application of the optimal strategy application policies proposed above may reduce the yearly unnecessary total KPI penalty by 88,000 (33%) compared to the lightest compulsory corrective strategy application policy without preventive strategies. For the models discussed in this paper, this percentage is 32% (for the model with paired coated rollers) and 39% (for the model with a standalone uncoated roller).

## 4. Conclusions, Limitations and Future Work

This work demonstrated that the DSF Core is a generic, but also complex, algorithmic component, which finds the optimal lifetime extension strategy application policy for industrial equipment, considering various dependencies among machine components and their stop types. It relies on simulation to evaluate the mean total KPI (dependent on the economic and environmental factors using the MCDA method along with an automatic objective weighting method) for each tested policy within a large time interval covering the life cycle of the production line and finds a near-optimal policy with only a few evaluations of the objective function, also considering the number of variables to be optimized by carefully updating their values. The optimization starts from the lightest compulsory corrective strategy application policy without preventive strategies to avoid searching very far from the optimal and simplest solution possible. Indeed, there is usually no unique optimal policy. In the first phase of training, the optimal corrective strategies are found, still assuming that no preventive strategies take place, and, in the second phase, the optimization continues by updating the decision variables related to the preventive policy. Based on the above, the DSF Core enables direct comparison of the considered KPIs among the initial, (optimal) corrective and final solution. The conclusion was that there is margin for KPI improvement for all three pilots discussed in this paper, by 30–40% approximately. Of course, these KPIs may be further improved (or have already been improved) for every pilot by third-party algorithmic services discussed above, as well as thanks to the direct real-time monitoring of the accurate sensorial data and the software-irrelevant activities performed at the pilots.

Regarding the limitations of the work, as mentioned above, the time complexity of each simulation is quadratic with respect to the number of stop types. In addition, it is linear with respect to the number of timestamps. Between the two optimization phases, the most time-consuming is the second one because the candidate values per variable to be optimized are more, and the number of variables is higher. The algorithm can find a near-optimal solution with few function evaluations in each of the two optimization phases (about 2MJ with respect to the case during the second phase, where *M* is the number of variables to be optimized and *J* is the number of epochs during that phase, and M≤23, J≤4 for the models trained). However, each individual function evaluation corresponds to a simulation of the production line operation during an interval large enough to include tens of instances for (almost) each stop type and with a sampling step not much higher than the shortest modelled mean stop duration of the specific stop type. These two conditions should be met so the numbers of stops and their downtime impact may be estimated accurately enough. Since about 1000 timestamps were processed per second during the simulation, the whole training usually takes hours or a few days. The memory complexity is linear with respect to both the number of components and the number of timestamps. Most of the trained models do not involve memory concerns, but the most complex models for ZORLUTEKS, that is, those involving motors with two rollers, consume significant memory. Although memory could be reduced with a small adaptation of the algorithm, this would lead to a further increase of execution time, so this idea was not implemented. The simulation approach may be more time- and memory-complex than an analytic solution in a simple case, but it is more generic and easily scalable.

The DSF Core is going to be deployed at the above pilot locations. The integration with the other relevant algorithms is ongoing as well. After deployment, the benefit offered by the DSF Core to the pilots will be evaluated. As future research work, the plan is to find the fastest but equally generic KPI evaluation methods that will avoid a significant part of the calculations during a normal simulation.

## Figures and Tables

**Table 1:** Relation among data, KPIs, decisions and methods based on bibliography about decision support and optimization plans in manufacturing.

Input Data	Optimization Criteria	Decisions	Methods	Reference(s)
-	economic (costs, profit, net present value…), environmental (greenhouse gas, energy, life-cycle-assessment-based, water, waste,…), other (service level, social)	decisions for green supply chains about production–distribution planning, inventory management	multi-criteria decision methodology (MCDM) [analytic hierarchy process (AHP), analytic network process, decision-making trial and evaluation laboratory, elimination and choice expressing reality, preference ranking organization method for enrichment of evaluations, technique for order of preference by similarity to ideal solution, utility additive, ϵ-constraint, goal programming, weighting method] (+interpretive structural modelling)	[23,24]
product performance, historical product design, customer demands, assembly requirements, environment impacts,…	-	intelligent product and service design	data mining, artificial intelligence, big data analysis (deep learning on diverse input)	[25]
{historical faults, product quality} → {failure forecasting, products lifetime}	-	intelligent production (predictive maintenance planning,…)		
material delivery, energy (maybe predicted by process variables)	energy efficiency (of shop-floor material handling)	intelligent production (material route),…		
product operation status → equipment performance, product quality monitoring, historical faults, customer evaluation	-	intelligent maintenance and service (customer service, product support, maintenance)		
product life cycle history ({product design index, maintenance history, operation status,…} → {remaining lifetime, degradation status}, environmental factors,…)	environmental	intelligent recovery (reuse, remanufacturing, repair, recycling, disposal,…)		
linking equipment and process data to inspection and metrology data	product quality and yield	-		
logistics data	shop-floor logistics (productivity, delivery time)	-		
-	environmental,…	design	ISO 20140 (2013) → classification, environmental effect ISO 10303e1 (1994) → product geometry, material choice	[17]
proportion of each item obtained after disassembly of 1 unit of its parent fixed setup cost for disassembly of each parent item in each period unit holding cost for each item in each period demand of each leaf item in each period	costs (setup, penalty, overload, lost sales, inventory holding)	disassembly lot-sizing (scheduling) disassembly quantity of each parent item in each period if each parent item will be disassembled in each period inventory level of each item at the end of each period	various	[26]
unit production cost for (re)manufacturing in each period fixed setup cost for (re)manufacturing in each period unit holding cost for serviceable products/returns in each period demand for serviceable products in each period quantity of returns in each period	production, setup, holding costs associated to (re)manufacturing	from product to raw material recycling production quantity for (re)manufacturing in each period if (re)manufacturing will occur in each period inventory level of serviceable products/returns in each period		
production coefficient of each co-product unit production cost of each co-product in each period fixed setup cost in each period unit holding cost for each co-product in each period demand of each co-product in each period	setup, production, inventory costs	by-products vs. co-products production quantity in each period if production will occur in each period inventory level of each co-product in each period		
unit production cost/emission in each period fixed setup cost/emission in each period unit holding cost/emission in each period demand for products in each period global emission capacity	costs	greenhouse gas emissions and energy consumption production quantity in each period if production will occur in each period inventory level in each period		
failure probability threshold (determining replacement decision)	replacement/ maintenance cost	replacement	gas turbine measurements → failure probability (supervised classification with fuzzy unordered rule induction algorithm)	[27]
-	environmental, economic, social	material alternatives in manufacturing	multi-criteria decision analysis (MCDA)	[21]
data from a local control drive remanufacturing company	technological, economic, resource utilization, environmental metrics	- (only a continuous remanufacturability score is the output)	fuzzy inference system	[28]
disassembly cost (dependent on disassembly labor, labor rate, tooling and material costs, overheads) net recoverable value (NRV) (dependent on component value, and processing, collection and disassembly costs) EoL impact (EOLI) (weighted sum of component-specific impacts)	environmental (EOLI, CO_2_-SO_2_ emissions, energy), economic [NRV, logistic–disassembly cost, product cost (incineration, recycling, landfill)], societal (number of employees and their exposure to hazardous materials)	remanufacturing, reconditioning, refurbishment, cannibalization, repair, recycling	mathematical optimization, MCDM (selected in the respective paper), empirical methods	[29]
-	working status, quality, disassemblability, cleanability, repair–replacement ability, spare parts availability, market for recovered 2nd-hand products, green design and hazardous waste	reuse/resell, product upgrade (repair, remanufacturing, refurbishment), materials recovery (cannibalization, recycling), disposal	fuzzy logic	[30]
item useful life time [31,32], technology/design cycle [33,34,35], wear-out life [33,35], standard or interchangeable item [36,37], number of components [33], product architecture and level of integration [33,34], disassembly effort [38,39,40], materials separability [34], investment costs [41,42], recovery process cost [43], new item value [34,44], used item value [34], lost sale in primary market [34,45], EoL product location [46], collection cost [34,41,42], demand volume [41], cost of legal compliance [47], regulations on recycled quota [48], energy yield [49], material yield [50], liquid and solid waste impact [50], air emissions [49,50], hazardous material contents [51], reason for discard, purpose of ownership, consumer opinion toward used product [43], damages/benefit to human health [52,53], society involvement in recovery programs [54], green party pressure [55], fuel cell cost data [for end and bipolar plates, membrane electrolyte assembly (MEA) [gas diffusion layer (GDL), anode and cathode catalyst, membrane], gaskets, current collectors, electrical jumpers, bolts], disassembly time (to unplug electrical jumpers, unscrew end plate bolts, and remove end and bipolar plates, current collector, gasket, MEA assembly, GDLs, cathode and anode catalysts and membrane)	engineering (product, process), business (market, supply–demand, legal-political), environmental (resources, pollution), societal (targeted segment, overall society)	level 1: reuse, recycling, incineration level 2: degree of reuse/recycling/ incineration	exhaustive enumeration, mathematical optimization, multi-criteria (selected with AHP in the respective paper), clustering, empirical	[22]
Easy-LCA → emissions to air/water/soil: CO_2_, SO_x_, NO_x_, biochemical oxygen demand, {bill of materials, energy} → global warming potential [Eco-indicator 99 inapplicable to Asia] QFDNavi/LCPlanner → QFD data [quantitative quality characteristics of the target product (product weight,…), components composing the product, and their importance] → cost importance analysis	cost, environmental, quality	upgrade/maintenance, product/component reuse, material recycling, disposal	quality function deployment (QFD) (transforms qualitative user demands into quantitative parameters,…)	[18]
recovery fraction (percentage of environmental impact corresponding to weight of reusable components at j-th recovery cycle) useful life (extended by recovery processes)	environmental effectiveness of recovery cycles (intensity of resource use in terms of extension of the product’s useful life)	design alternative	comparison among few alternatives unitary indicators based on Eco-indicator 95 method (Goedkoop 1995)	[56]
if each unit/distribution hub/retailer is open per period capacities of each supplier/unit/distribution hub/retailer stake per raw material per part, stake per part per product demand per product per customer per period per scenario opening cost for each unit/distribution hub/retailer (fixed) unit cost of purchasing/processing/assembling/ sorting and packing/dismantling/disposal/ reprocessing (uniform)/shipping (/km) percentages of collected demand per retailer per customer per scenario percentages of disassembled amounts disposed/resent to units	total supply chain cost	quantities shipped among supplier, processing/assembling/ reprocessing/sorting and dismantling/disposal units, distribution hubs, retailers, customers	mixed integer linear programming (selected in respective paper), fuzzy logic, branch and bound, spanning tree and prufer number, stochastic programming, goal programming	[57]
expected (based on Markov chain) frequency of accepting products for remanufacturing/rejecting disposed products/disposing products due to storage capacity limits/customer order completion delays/storing recoverable products/discarding products during remanufacturing, expected revenue from remanufacturing a returned product [quality follows normal, exponential or beta (in respective paper) distribution], salvage cost, cost of recoverable products inventory establishment, cost of customer order completion delay, holding cost of returned products, cost of discarding recoverable product during remanufacturing (fixed unit costs)	profit	optimal minimum quality to accept into remanufacturing facility and quantity of parts to purchase from external suppliers, recoverable products inventory capacity	mixed integer non-linear programming (MINLP), queueing model, continuous time Markov chain, quasi-birth–death process, matrix-geometric method	[58]
operational cost (remanufacturing, product disassembly, part refurbishing, inventory holding, product and part inventories) purchasing and under-stocking cost (returns acquisition and inspection, purchasing from external suppliers, total under-stocking) set up and idle cost (set-up cost of remanufacturing, disassembly, refurbishing sites as well as their idle costs) revenue (by selling remanufactured products into the secondary market) (fixed unit costs – beta-distributed quality)	total profit of facility	optimal minimum quality to remanufacture, sales, quantity of purchased/disassembled/ remanufactured/refurbished/ disposed products/parts, inventory levels, binary variables for setup of remanufacturing/disassembly/ refurbishing	MINLP → quadratic mixed integer programming	[59]
quality of product (or subassembly, or component) → revenue (health state of product, its parts and sub-parts considered as random variables) remaining usage potential (RUP) of a subassembly or component revenue generated by a subassembly or component (function of RUP) cost of disassembly task (fixed) processing time of task (fixed, should have been considered as variable) cost per disassembly time unit (fixed)	disassembly profit (revenue by recovered parts – disassembly costs)	disassembly alternatives (level) in remanufacturing	quality modeled using RUP [considered as normal distribution truncated in [0,1]]	[60]
production cost (sum of purchasing, manufacturing and assembly costs) CO_2_ emissions per purchased/manufactured/assembled unit customer demand, due date, supplier/manufacturer capacity, lot-size release and machine yield → lead time (purchasing new+recycled) (fixed parameters – uncertainty faced by FMOLP)	costs, CO_2_ emissions, lead time	lot size (units) per component purchased/released per manufacturing/assembling machine	fuzzy multi-objective linear programming model (FMOLP)	[61]

**Table 2:** Relation among data, cost types and methods based on bibliography about cost analysis and cost modelling tools.

Input Data	Cost Types	Methods	Reference(s)
three-dimensional model and related information (materials, tolerances, surface finish,…) geometry, manufacturing process, materials, other non-geometric information	manufacturing	predictive analytics modelling, least absolute shrinkage and selection operator and elastic net modelling ^1^, process-oriented feature extraction	[62]
raw material price, initial investment	labor, part material, energy, support material, overheads	fixed values of parameters	[63]
few primary user parameters, optional secondary user parameters to increase estimation accuracy	machine, material, labor, post-processing	break-down (build time estimated by considering activities undergone by machine for preparation of a layer and multiplying it by total number of layers)	[64]
module dimensions and format, factory assumptions and cost inputs, production equipment assumptions, materials	manufacturing (sum of tool and facility, spare parts, footprint, electricity, material cost-usage)	Monte Carlo analysis to estimate cost distribution	[65]
product type, design method, machining condition parameters (tool diameter/tip, cutting/free movement feed rate, cut depth, step over)	machining, printing (process), material, labor, post-processing	calculating parameters affecting air manifold production price (cost types) (machining simulations performed with “Power mill 2018 software”)	[66]
vendor’s/buyer’s holding cost, cost of replacing imperfect goods, cumulative distribution function, mean/variance of lead time, buyer’s ordering cost, backorder cost, coefficient of variance per time period, batch size, initial probability of shifting out-of-control from in-control state, vendor’s initial setup cost, demand scaling parameter, scaling/shape parameters related to advertising demand function, annual fractional cost of capital investment, variation constant of product tool/die cost, machine running cost, scaling parameters related to PQI/SCR, reorder point for buyer, expected backorder quantity/on-hand inventory, mean lead time demand	vendor [holding, setup, variable production, defective, process quality improvement (PQI), setup cost reduction (SCR), advertisement]	exact computations also considering assumptions based on bibliography	[67]

^1^ These two are regularization and are dimensionality reduction techniques for linear regression.

**Table 3:** ETTF distribution parameters and METTF for the spraying cabin (GORENJE), ignoring the already occurring lifetime extension thanks to time-based inspection.

Component	Scale (η)	Shape (β)	METTF
Power supply unit	144 D = 34.3 woD	2	128 D = 30.4 woD
Pump	4.8 years = 1.1 working years = 417 woD	2	4.3 years = 370 woD
Other	33 D = 7.9 woD	2	29 D = 7.0 woD

**Table 4:** Direct costs (EUR) during strategies for the spraying cabin (GORENJE).

Component	Strategy	Gross	Net
Power supply unit	Maintenance	257.209375	220
	Replacement	3334.03125	3210
Pump	Maintenance	76.0078125	45
	Replacement	840.8140625	785
Hose	Replacement	146.0078125	115
Spraying gun	Maintenance	181.0078125	150
	Replacement	3686.0078125	3655

**Table 5:** KPIs yielded from training the model for the spraying cabin (GORENJE).

		Economic (EUR/Year)		Scrap (Discarded Parts/Year)		Total	
**Ideal**	**Per Component**	−45,542		62.167		−45,076	
	**Total**	−182,168		248.667		−180,303	
	**Component**	**Actual**	**Extra**	**Actual**	**Extra**	**Actual**	**Extra**
**Initial = corrective solution**	Hose	−39,639	5903	57.086	−5.081	−39,211	5865
	Power supply unit	−40,965	4577	57.086	−5.081	−40,537	4539
	Pump	−41,799	3743	57.086	−5.081	−41,371	3705
	Spraying gun	−39,080	6462	57.086	−5.081	−38,652	6424
	Total	−161,482	20,686	228.343	−20.323	−159,770	20,533
**Final solution**	Hose	−41,412	4130	60.474	−1.692	−40,958	4118
	Power supply unit	−43,446	2096	60.474	−1.692	−42,992	2084
	Pump	−44,281	1261	60.474	−1.692	−43,827	1248
	Spraying gun	−40,735	4807	60.474	−1.692	−40,281	4795
	Total	−169,873	12,295	241.897	−6.770	−168,059	12,244

**Table 6:** Numbers of stop instances yielded from training the model for the spraying cabin (GORENJE) within the simulation interval (200 years). Underlined and italic contents correspond to failures and strategies, respectively.

Component	Stop Type	Initial = Corrective Solution	Final Solution
Hose	Failure	2464	1275
	*Replacement*	*2464*	*3726*
Power supply unit	Failure	665	666
	*Maintenance*	*665*	*666*
	*Replacement*	*0*	*0*
Pump	Failure	56	56
	*Maintenance*	*56*	*56*
	*Replacement*	*0*	*0*
Spraying gun	Failure	2585	1436
	*Maintenance*	*2585*	*3726*
	*Replacement*	*0*	*0*

**Table 7:** Production time percentage for every component yielded from training the model for the spraying cabin (GORENJE).

Ideal	Initial = Corrective Solution	Final Solution
23.8095%	23.6471%	23.7553%

**Table 8:** Total KPIs with respect to components with summation for the whole production line, yielded from training the model for GORENJE.

		Economic (EUR/Year)		Scrap (Discarded Parts/Year)		Total (Yearly)	
	**Model**	**Actual**	**Extra**	**Actual**	**Extra**	**Actual**	**Extra**
**Initial = corrective solution**	Quartet (×12)	−161,482	20,686	228.343	−20.323	−159,770	20,533
	Total	−1938 K	248,229	2740.12	−243.88	−1917 K	246,400
**Final solution**	Quartet (×12)	−169,873	12,295	241.897	−6.770	-168,059	12,244
	Total	−2038 K	147,542	2902.76	−81.24	−2017 K	146,933

**Table 9:** Stops per component of the friction welding machine (HWH).

Component	Failures	Strategies
Static	single failure type	refurbishment
Motor	mechanical fatigue ^1^, lubricant	maintenance ^2^, replacement
Spindle	mechanical fatigue, lubricant	maintenance, replacement
Sample-holder	single failure type	replacement
Sample-detector	single failure type	replacement

^1^ This is bearing mechanical fatigue for both the motor and the spindle. ^2^ This coincides with lubrication for both the motor and the spindle

**Table 10:** ETTF distribution parameters and METTF for the friction welding machine (HWH).

Component	Failure Type	Scale (η)	Shape (β)	METTF
Static	Failure	1179.02 woD	127.53015316411776 (so that CV = 0.01) ^1^	18 years ^2^ = 1173.75 woD
Motor/Spindle	Mechanical fatigue	24.9 years = 4.45 working years = 1625.36 woD	1.5	22.50 years = 1467.29 woD
	Lubricant	5 years = 1826.25 D	2	4.43 years = 1618.47 D
Sample-holder	Failure	150,000 parts = 3 years = 195.63 woD	3	2.68 years = 174.69 woD

^1^ Such a low CV value was selected to avoid high uncertainty in evaluation, since static failures rarely happen and imply high costs from the failures themselves and the compulsory subsequent refurbishment and motor–spindle replacement strategies. ^2^ Since an 8-year extension of a 10-year old machine was expected.

**Table 11:** Direct costs (EUR) during strategies for the friction welding machine (HWH).

Component	Strategy	Duration-Dependent (Gross)	Duration-Independent	Total (Gross)	Total (Net)
Static	Refurbishment	not studied	not studied	21,730	21,534.7
Motor	Maintenance	223.2	1070	1293.2	1097.9
	Replacement	223.2	3120	3343.2	3147.9
Spindle	Maintenance	223.2	1060	1283.2	1087.9
	Replacement	223.2	5810	6033.2	5837.9
Sample-holder	Replacement	27.9	101	128.9	128.9
Sample-detector	Replacement	27.9	121	148.9	148.9

**Table 12:** KPIs (EUR/year) yielded from training the model for the friction welding machine (HWH).

Component	Ideal	Initial = Corrective Solution		Final Solution	
		Actual	Extra	Actual	Extra
Motor	−99,094	−97,301	1793	−97,695	1398
Sample-detector	−99,094	−97,931	1162	−98,166	927
Sample-holder	−99,094	−98,336	758	−98,870	223
Spindle	−99,094	−96,314	2780	−97,317	1776
Static	−99,094	−97,801	1293	−97,801	1293
Total	−495,468	−487,682	7786	−489,850	5618

**Table 13:** Numbers of stop instances yielded from training the model for the friction welding machine (HWH) within the simulation interval (292 years). Underlined and italic contents correspond to failures and strategies, respectively.

Component	Stop Type	Initial = Corrective Solution	Final Solution
Motor	Lubricant	46	29
	Mechanical fatigue	60	38
	*Maintenance*	*46*	*82*
	*Replacement*	*76*	*54*
Sample-detector	Failure	158	14
	*Replacement*	*158*	*1631*
Sample-holder	Failure	104	16
	*Replacement*	*104*	*258*
Spindle	Lubricant	57	28
	Mechanical fatigue	66	34
	*Maintenance*	*57*	*82*
	*Replacement*	*82*	*50*
Static	Failure	16	16
	*Refurbishment*	*16*	*16*

**Table 14:** Stops per component of the bleaching machine (ZORLUTEKS) involved in the relevant models discussed in this paper.

Component	Failures	Strategies
Roller coating	single failure type	maintenance (=grinding), replacement
Double bearing-lubricant pair	left bearing mechanical fatigue, right bearing mechanical fatigue, left bearing lubricant, right bearing lubricant	maintenance (=lubricant replacement for both bearings), replacement
Motor	complete, lubricant	maintenance (lubrication and winding alternatives), replacement

**Table 15:** Production metrics (2020–2021) for the bleaching machine (ZORLUTEKS).

Operation	Mean Output Fabric Area (Mm${}^{2}$/Year)			Time Percentage (%)		
	Bleaching	Washing	Total	Bleaching	Washing	Total
Route	27.72	0.34	28.06	54.28	0.72	55.00
Repair	3.33	1.63	4.96	7.55	4.25	11.80
Sample	0.00	0.00	0.00	0.01	0.00	0.01
Total	31.05	1.97	33.02	61.84	4.97	66.81

**Table 16:** Time to recover from roller coating (appeared as “roller and shaft” in the stop dataset), double bearing-lubricant pair (appeared as “bearing and mounting guide” in the stop dataset) and motor failures in the bleaching machine (ZORLUTEKS).

Component	Mean (min)	CV	Weibull Scale (min)	Weibull Shape
Roller coating	65.031579	1.8217826	41.612199	0.5832355
Double bearing-lubricant pair	53.293333	2.9996844	17.292100	0.4113669
Motor	31.915254	0.4667525	36.030221	2.2688522

**Table 17:** ETTF distribution parameters and METTF for the models discussed in this paper for the bleaching machine (ZORLUTEKS).

Component	Failure Type	Scale (η)	Shape (β)	METTF
Roller coating	Failure	2 years = 730.5 D	3	1.785959 years = 652.3215 D
Double bearing-lubricant pair	Mechanical fatigue of particular bearing (model with paired coated rollers)	7 years = 2556.75 D	1.5	6.319217 years = 2308.094 D
	Mechanical fatigue of particular bearing (model with standalone uncoated roller)	4.5 years = 1643.625 D	1.5	4.062354 years = 1483.775 D
	Lubricant for particular bearing	3 years = 1095.75 D	^1^ 15	2.896992 years = 1058.126 D
Motor	Complete (model with paired coated rollers)	3 years = 1095.75 D	1.5	2.708236 years = 989.1832 D
	Complete (model with standalone uncoated roller)	2 years = 730.5 D	1.5	1.805491 years = 659.4554 D
	Lubricant	4 years = 1461 D	3	3.571918 years = 1304.643 D

^1^ In the respective degradation model *β* = 3 was defined, but since it is assumed that lubricant failure in a particular bearing-lubricant pair may be well-forecast thanks to vibration anomalies detection, the value was increased to reduce the variance of the distribution.

**Table 18:** Direct costs (EUR) during strategies for every roller coating, double bearing-lubricant pair and motor of the bleaching machine (ZORLUTEKS).

Component	Strategy	Gross	Net
Roller coating	Maintenance	1307	175
	Replacement	1582	450
Double bearing-lubricant pair	Maintenance	6	6
	Replacement	1458	326
Motor	Maintenance (lubrication)	21	21
	Maintenance (winding)	1282	150
	Replacement	4132	3000

**Table 19:** Direct failure costs for every failure type of roller coating, double bearing-lubricant pair and motor of the bleaching machine (ZORLUTEKS).

Component	Bad Whiteness Probability	Expected Waiting Time Due to Bad Whiteness	Expected Gross Profit Loss Due to Bad Whiteness
Roller coating	41%	9.4 min	EUR 464
Double bearing-lubricant pair	43%	9.9 min	EUR 487
Motor	14%	3.2 min	EUR 159

**Table 20:** Optimal corrective strategies for the model with paired coated rollers for the bleaching machine (ZORLUTEKS).

Component	Failure Type	Corrective Strategy
Coating of any roller	Failure	replacement
Double bearing-lubricant pair of any roller	Left bearing lubricant	maintenance
	Left bearing mechanical fatigue	replacement
	Right bearing lubricant	maintenance
	Right bearing mechanical fatigue	maintenance
Motor	Complete	maintenance (winding)
	Lubricant	maintenance (lubrication)

**Table 21:** KPIs yielded from training the model with paired coated rollers for the bleaching machine (ZORLUTEKS).

		Economic (EUR/Year)		Energy (kWh/Year)		Other Environmental (kg/Year)		Total (Yearly)	
**Ideal**	**Per Component**	**−206,863**		**3355**		**276,163**		**−200,696**	
	**Total**	**−103,4314**		**16,775**		**1,380,814**		**−1,003,478**	
	**Component**	**Actual**	**Extra**	**Actual**	**Extra**	**Actual**	**Extra**	**Actual**	**Extra**
**Initial solution**	Coating of bottom roller	−199,778	7084	3269	−86	269,093	−7070	−193,769	6927
	Coating of top roller	−199,761	7102	3269	−86	269,093	−7070	−193,751	6944
	Double bearing-lubricant pair of bottom roller	−200,623	6240	3269	−86	269,093	−7070	−194,614	6082
	Double bearing-lubricant pair of top roller	−200,628	6235	3269	−86	269,093	−7070	−194,619	6077
	Motor	−200,889	5974	3269	−86	269,093	−7070	−194,880	5816
	Total	−1.002 M	32,635	16,346	−429	1.345 M	−35,349	−971,632	31,846
**Corrective solution**	Coating of bottom roller	−200,199	6664	3278	−77	269,854	−6309	−194,173	6523
	Coating of top roller	−200,148	6715	3278	−77	269,855	−6308	−194,122	6574
	Double bearing-lubricant pair of bottom roller	−201,406	5457	3278	−77	269,824	−6339	−195,381	5315
	Double bearing-lubricant pair of top roller	−201,387	5476	3278	−77	269,824	−6339	−195,361	5335
	Motor	−201,415	5448	3278	−77	269,820	−6342	−195,389	5306
	Total	−1.005 M	29,759	16,390	−385	1.349 M	−31,637	−974,425	29,053
**Final solution**	Coating of bottom roller	−202,717	4145	3297	−58	271,432	−4731	−196,656	4040
	Coating of top roller	−202,686	4177	3297	−58	271,434	−4729	−196,621	4075
	Double bearing-lubricant pair of bottom roller	−202,391	4472	3297	−58	271,426	−4737	−196,326	4370
	Double bearing-lubricant pair of top roller	−202,351	4512	3297	−58	271,426	−4737	−196,286	4410
	Motor	−202,084	4779	3297	−58	271,425	−4738	−196,019	4677
	Total	−1.012 M	22,084	16,487	−288	1.357 M	−23,672	−981,922	21,556

**Table 22:** Numbers of stop instances yielded from training the model with paired coated rollers for the bleaching machine (ZORLUTEKS) within the simulation interval (200 years). Underlined and italic contents correspond to failures and strategies, respectively.

Component	Stop Type	Initial Solution	Corrective Solution	Final Solution
Coating of bottom roller	Failure	202	187	47
	*Maintenance*	*202*	*0*	*113*
	*Replacement*	*0*	*187*	*47*
Coating of top roller	Failure	204	192	57
	*Maintenance*	*204*	*0*	*104*
	*Replacement*	*0*	*192*	*57*
Double bearing-lubricant pair of bottom roller	Left bearing lubricant	5	20	0
	Left bearing mechanical fatigue	204	50	33
	Right bearing lubricant	1	23	0
	Right bearing mechanical fatigue	173	46	28
	*Maintenance*	*383*	*89*	*3291*
	*Replacement*	*0*	*50*	*33*
Double bearing-lubricant pair of top roller	Left bearing lubricant	2	21	0
	Left bearing mechanical fatigue	184	51	33
	Right bearing lubricant	4	17	0
	Right bearing mechanical fatigue	191	55	34
	*Maintenance*	*381*	*93*	*3259*
	*Replacement*	*0*	*51*	*33*
Motor	Complete	87	90	75
	Lubricant	57	54	6
	*Maintenance (lubrication)*	*57*	*54*	*3025*
	*Maintenance (winding)*	*87*	*90*	*75*
	*Replacement*	*0*	*0*	*0*

**Table 23:** Production time percentage for every component yielded from training the model with paired coated rollers for the bleaching machine (ZORLUTEKS).

Ideal	Initial Solution	Corrective Solution	Final Solution
100.0000%	99.7490%	99.7748%	99.8318%

**Table 24:** Optimal corrective strategies for the model with standalone uncoated roller for the bleaching machine (ZORLUTEKS).

Component	Failure Type	Corrective Strategy
Double bearing-lubricant pair	Left bearing lubricant	replacement
	Left bearing mechanical fatigue	replacement
	Right bearing lubricant	replacement
	Right bearing mechanical fatigue	maintenance
Motor	Complete	maintenance (winding)
	Lubricant	maintenance (lubrication)

**Table 25:** KPIs yielded from training the model with standalone uncoated roller for the bleaching machine (ZORLUTEKS).

		Economic (EUR/Year)		Energy (kWh/Year)		Other Environmental (kg/Year)		Total (Yearly)	
**Ideal**	**Per Component**	−206,863		3355		276,163		−200,696	
	**Total**	−413,725		6710		552,325		−401,391	
	**Component**	**Actual**	**Extra**	**Actual**	**Extra**	**Actual**	**Extra**	**Actual**	**Extra**
**Initial solution**	Double bearing-lubricant pair	−202,088	4774	3301	−54	271,717	−4446	−196,020	4675
	Motor	−202,611	4251	3301	−54	271,717	−4446	−196,544	4152
	Total	−404,700	9026	6602	−108	543,434	−8891	−392,564	8827
**Corrective solution**	Double bearing-lubricant pair	−203,472	3390	3318	−37	273,083	−3080	−197,374	3322
	Motor	−203,643	3220	3318	−37	273,077	−3086	−197,544	3151
	Total	−407,115	6611	6635	−75	546,160	−6165	−394,918	6473
**Final solution**	Double bearing-lubricant pair	−204,185	2678	3323	−32	273,512	−2650	−198,077	2619
	Motor	−204,001	2862	3323	−32	273,509	−2654	−197,893	2803
	Total	−408,186	5540	6646	−64	547,021	−5304	−395,970	5421

**Table 26:** Numbers of stop instances yielded from training the model with standalone uncoated roller for the bleaching machine (ZORLUTEKS) within the simulation interval (200 years). Underlined and italic contents correspond to failures and strategies, respectively.

Component	Stop Type	Initial Solution	Corrective Solution	Final Solution
Double bearing-lubricant pair	Left bearing lubricant	2	23	1
	Left bearing mechanical fatigue	298	64	55
	Right bearing lubricant	0	10	0
	Right bearing mechanical fatigue	286	55	45
	*Maintenance*	*586*	*55*	*1184*
	*Replacement*	*0*	*97*	*56*
Motor	Complete	121	119	117
	Lubricant	55	57	31
	*Maintenance (lubrication)*	*55*	*57*	*61*
	*Maintenance (winding)*	*121*	*119*	*117*
	*Replacement*	*0*	*0*	*0*

**Table 27:** Production time percentage for every component yielded from training the model with standalone uncoated roller for the bleaching machine (ZORLUTEKS).

Ideal	Initial Solution	Corrective Solution	Final Solution
100.0000%	99.9369%	99.9562%	99.9623%

**Table 28:** Total KPIs with respect to components with summation for the whole production line, yielded from training all models for ZORLUTEKS.

		Economic (EUR/Year)		Energy (kWh/year)		Other Environmental (kg/Year)		Total (Yearly)	
	**Model**	**Actual**	**Extra**	**Actual**	**Extra**	**Actual**	**Extra**	**Actual**	**Extra**
**Initial solution**	Motor of type 2_2 (×5)	−1.000 M	34236	16323	−452	1.344 M	−37,219	−970,074	33,404
	Motor of type 2_3_7	−1.002 M	32,635	16,346	−429	1.345 M	−35,349	−971,632	31,846
	Motor of type 2_3_4.5	−994,133	40,180	16,251	−524	1.338 M	−43,150	−964,261	39,217
	Motor of type 1 (×2)	−404,700	9026	6602	−108	543,434	−8891	−392,564	8827
	Motor of type 0 (×2)	−202,585	4277	3302	−53	271,766	−4396	−196,516	4179
	Inverter of type 9 (×6)	−206,686	177	3352	−2.6	275,947	−216	−200,524	172
	Inverter of type 10 (×4)	−206,742	121	3353	−1.8	276,017	−145	−200,578	118
	Total	−10.28 M	272 K	167.5 K	−3558	13.79 M	−293 K	−9.97 M	266 K
**Corrective solution**	Motor of type 2_2 (×5)	−1.002 M	31,900	16,363	−412	1.347 M	−33,862	−972,335	31,143
	Motor of type 2_3_7	−1.005 M	29,759	16,390	−385	1.349 M	−31,637	−974,425	29,053
	Motor of type 2_3_4.5	−1.004 M	30,275	16,383	−392	1.349 M	−32,196	−973,923	29,556
	Motor of type 1 (×2)	−407,115	6611	6635	−75	546,160	−6165	−394,918	6473
	Motor of type 0 (×2)	−202,585	4277	3302	−53	271,766	−4396	−196,516	4179
	Inverter of type 9 (×6)	−206,686	177	3352	−2.6	275,947	−216	−200,524	172
	Inverter of type 10 (×4)	−206,742	121	3353	−1.8	276,017	−145	−200,578	118
	Total	-10.31 M	243 K	168.0 K	−3116	13.83 M	−256 K	−10.0 M	237 K
**Final solution**	Motor of type 2_2 (×5)	−1.011 M	23,197	16,473	−302	1.356 M	−24,840	−980,836	22,642
	Motor of type 2_3_7	−1.012 M	22,084	16,487	−288	1.357 M	−23,672	−981,922	21,556
	Motor of type 2_3_4.5	−1.010 M	23,864	16,467	−308	1.355 M	−25,341	−980,180	23,298
	Motor of type 1 (×2)	−408,186	5540	6646	−64	547,021	−5304	−395,970	5421
	Motor of type 0 (×2)	−202,790	4073	3304	−51	271,967	−4195	−196,717	3979
	Inverter of type 9 (×6)	−206,723	139	3353	−2.1	275,993	−169	−200,560	136
	Inverter of type 10 (×4)	−206,742	121	3353	−1.8	276,017	−145	−200,578	118
	Total	−10.37 M	182 K	168.7 K	−2356	13.89 M	−194 K	−10.1 M	178 K

## Data Availability

All input parameters and specifications needed to train the DSF Core models discussed in this paper have already been provided within the main part of the manuscript with the permission of the pilots.

## Abbreviations

The following abbreviations are used in this manuscript:

AHP	analytic hierarchy process
CAGR	compound annual growth rate
CO_2_	carbon dioxide
CV	coefficient of variation
DSF	Decision Support Framework
Easy-LCA	name of software mentioned in [18]
EoL	End of Life
EOLI	EoL impact
ETTF	Equivalent Time To Failure
FMOLP	fuzzy multi-objective linear programming model
GDL	gas diffusion layer
ISO	International Organization for Standardization
KPI	Key Performance Indicator
LCA	Life Cycle Assessment
LCC	Life Cycle Cost
LCPlanner	name of software mentioned in [18]
MCDA	multi-criteria decision analysis
MCDM	multi-criteria decision methodology
MEA	membrane electrolyte assembly
METTF	Mean ETTF
MINLP	mixed integer non-linear programming
MRO	Maintenance, Repair and Overhaul
MSD	mean stop duration
MTTF	Mean Time To Failure
NO_x_	any mono-nitrogen oxide
NRV	net recoverable value
Ph	person hour
PQI	process quality improvement
QFD	quality function deployment
QFDNavi	name of software mentioned in [18]
RUP	remaining usage potential
SCR	setup cost reduction
SO_x_	any sulphur oxide
weh	welding hours
woD	working days
woh	working hours
womin	working minutes
